# Effects of prenatal psychotherapies and psychosocial interventions on depressive symptoms, anxious symptoms and stress: a systematic review and network meta-analysis

**DOI:** 10.3389/fpsyt.2025.1624924

**Published:** 2026-01-28

**Authors:** Lanting Huo, Xingfeng Yu, Yujia Ma, Lei Yang, Xiaomei Li

**Affiliations:** 1 Faculty of Nursing, Health Science Center, Xi’an Jiaotong University, Xi’an, Shaanxi, China; 2 The Nursing Department, Shaanxi Provincial People’s Hospital, Xi’an, Shaanxi, China; 3 Department of Nursing, Chinese People’s Liberation Army General Hospital, Beijing, China

**Keywords:** psychotherapies and psychosocial interventions, systematic review, network meta-analysis, perinatal depressive symptoms, perinatal anxious symptoms, perinatal stress

## Abstract

**Background:**

Perinatal psychological disorders have a significant impact on maternal and neonatal health. While many psychotherapies and psychosocial interventions have been developed and empirically evaluated for the management of perinatal psychological symptoms, the efficacy of these interventions and the most effective intervention modality remain inconclusive.

**Aim:**

To assess and compare the efficacy of various perinatal psychotherapies and psychosocial interventions for depressive symptoms, anxious symptoms and stress through a comprehensive systematic review with network meta-analysis.

**Methods:**

Eleven English and Chinese electronic databases were searched from inception to 30 November 2024. The Revised Cochrane Risk of Bias tool for randomized trials was used to assess the quality of the evidence. Standard pairwise meta-analyses were conducted for direct comparisons using Review Manager 5.3, and network meta-analyses were performed using Stata 16 and ADDIS 1.16.5 for different types of interventions based on random-effects models.

**Design:**

Systematic review and network meta-analysis with qualitative and quantitative syntheses were performed to holistically investigate intervention effectiveness.

**Results:**

A total of 85 randomized controlled trials were included in the analysis, 65 of which provided available data for quantitative synthesis. The synthesized results suggest that prenatal psychotherapies and psychosocial interventions tend to be effective in reducing depressive symptoms, anxious symptoms and stress with standardized mean differences (SMDs) of -0.70, -0.81, and -1.05, respectively. Multicomponent interventions (SMD=-1.14), interpersonal psychotherapy (SMD=-0.75), cognitive behavioral therapy (SMD=-0.69), mindfulness-based interventions (SMD=-0.68), and psychoeducation (SMD=-0.14) significantly decreased depression symptoms. Acceptance and commitment therapy (SMD=-1.66), interpersonal psychotherapy (SMD=-1.24), counseling (SMD= -1.13), multicomponent interventions (SMD=-0.86), mindfulness-based interventions (SMD=-0.84), and cognitive behavioral therapy (SMD=-0.81) were effective in alleviating anxiety symptoms. Multicomponent interventions (SMD=-5.74), mindfulness-based interventions (SMD=-1.31), cognitive behavioral therapy (SMD=-1.03), and counseling (SMD=-0.82) appeared to be effective strategies for the management of perinatal stress.

**Conclusions:**

Prenatal psychotherapies and psychosocial interventions are effective in reducing depressive symptoms, anxious symptoms and stress. Specifically, multicomponent interventions, counseling, and mindfulness-based interventions are superior for alleviating the symptoms of depressive symptoms, anxious symptoms and stress, respectively. Cognitive behavioral therapy may be a reasonable option that could produce a beneficial effect on all of these prenatal psychological problems.

## 1 Introduction

Pregnancy represents a special period marked by significant physiological and psychological changes that increase women’s vulnerability to mental health challenges (1), particularly depressive symptoms, anxious symptoms and stress. Recent studies have estimated that approximately 15% of pregnant women suffer from prenatal depressive or anxious disorders (2, 3). These debilitating disorders negatively impact both the pregnant women and their babies in multidimensional ways (4). For pregnant women, these negative influences may be physical in nature, such as sleep disorders, loss of appetite, or fatigue; psychological in nature, such as sadness or persistent postpartum mental health concerns; or psychosocial in nature, such as reduced social interactions, an impaired ability to perform daily tasks, or a poor relationship with family members (5). Meanwhile, perinatal mental health disorders can negatively affect fetal development and tend to cause psychological disorders (6, 7), delayed cognitive development (8), and emotional and behavioral problems later in life (9, 10). Therefore, the early detection and management of perinatal emotional disorders are essential to perinatal healthcare.

Both pharmacological and non-pharmacological treatments have demonstrated efficacy in alleviating symptoms of perinatal mental health disorders (11). However, due to concerns about the potential risks associated with medication use during pregnancy, a large proportion of pregnant women prefer non-pharmacological approaches (12). Furthermore, several international clinical guidelines have highlighted the fundamental role of psychotherapies and psychosocial interventions, such as cognitive behavioral therapy (CBT), interpersonal psychotherapy (IPT), mindfulness-based interventions (MBIs), psychoeducation, and peer support in managing adverse mental health disorders among perinatal women (13, 14). These approaches have demonstrated significant efficacy, particularly for individuals with mild to moderate symptoms.

There is substantial evidence supporting the efficacy of prenatal psychotherapies and psychosocial interventions for perinatal psychological symptoms, although the effectiveness varies across different disorders (15–17). However, existing research is predominantly limited to pairwise comparisons between single interventions and controls in randomized controlled trials and traditional meta-analyses, leaving the comparative effectiveness of different prenatal psychotherapies and psychosocial interventions unknown. Therefore, there is an urgent need for research that addresses the critical knowledge gaps regarding the synthesized efficacy of diverse prenatal psychotherapies and psychosocial interventions in alleviating psychological symptoms and their comparative treatment effects.

Network meta-analysis enables the simultaneous comparison of multiple treatment options by combining direct evidence (head-to-head trials) and indirect evidence (comparisons of treatments across trials with a shared comparator) (18). This approach provides a comprehensive ranking of different interventions, thereby contributing to the development of validated, feasible, and evidence-based therapeutic strategies in clinical practice (19). In this study, we conducted a systematic review and network meta-analysis to evaluate the effects of different prenatal psychotherapies and psychosocial interventions on depressive symptoms, anxious symptoms and stress based on both direct and indirect comparisons. We also aimed to identify preferable options to inform clinical decision-making for the effective management of these disorders.

## 2 Methods

### 2.1 Protocol registration

This systematic review was conducted following the Preferred Reporting Items for Systematic Reviews and Meta-Analyses (PRISMA) reporting guidelines (20) and the Cochrane Handbook for Systematic Reviews of Interventions (21). The protocol was pre-registered in the International Prospective Register of Systematic Reviews (PROSPERO) database at the Centre for Reviews and Dissemination in the United Kingdom (reference identifier: CRD420250652183).

### 2.2 Eligibility criteria

Eligible studies were required to have the following features:

1. Population: pregnant women aged over 18 years.
2. Intervention: participants in the intervention groups had to have received different prenatal psychotherapies or psychosocial interventions, including CBT, IPT, MBIs, psychoeducation (with no limitations on educational topics), BA (behavioral activation), PST (problem-solving therapy), ACT (acceptance and commitment therapy), counseling, and others. There were no restrictions on the format of the intervention (individual/group, therapist/self-guided), the aim of the intervention (prevention/treatment), the delivery platform (website/mobile app), the frequency of the intervention, or its duration. Interventions had to be initiated during the prenatal period but could extend into the postpartum phase for follow-up.
3. Comparator: participants in the control group were allowed to receive any form of control regime, including routine care, a waitlist, a blank control, or an attention control.
4. Outcomes: the outcome measures had to include at least one of the following psychological disorders: depressive symptoms, anxious symptoms and stress.

Study designs: this review included only randomized controlled trials (RCTs) published in English or Chinese.

### 2.3 Search strategies

Eight English electronic bibliographic databases(PubMed, EMBASE, Web of Science, ProQuest Dissertations and Theses, Scopus, PsycINFO, CINAHL, and the Cochrane Central Register of Controlled Trials) and three Chinese electronic bibliographic databases (China National Knowledge Infrastructure, Wan Fang Database, and the VIP Database) were searched from their inception to 30 November 2024 using the pre-specified keywords. The detailed search strategies for the different databases are presented in **Supplementary Table 1**.

### 2.4 Study selection

All duplicated studies were removed using EndNote X20. Two reviewers screened the titles and abstracts independently. Subsequently, the two reviewers independently completed full-text screening, with any discrepancies resolved through consensus discussion or consultation with a third researcher.

### 2.5 Data extraction and management

Two reviewers extracted details of the included studies using a researcher-designed tool following the Cochrane Handbook for Systematic Reviews of Interventions (22). The extracted data encompassed two main categories: (1) general study characteristics, including country, study design, participants’ week of gestation and age, intervention approach, sample size, assessment instruments, outcomes of interest, intention-to-treat analysis, and missing data management; and (2) intervention-specific details, including intervention aims, guidance methods, program outlines, delivery approach (whether or not digital platforms were used), and treatment regimens (number of sessions, duration, frequency, intervention period, and follow-ups).

### 2.6 Risk of bias assessment

After data extraction, the Cochrane Collaboration risk-of-bias tool was used to assess the quality of the included studies (23). Selection, performance, detection, attrition, and reporting biases were assessed using a series of signaling questions regarding random sequence generation, allocation concealment, participant and personnel blinding, outcome assessment blinding, incomplete outcome data, and selective reporting. The risk for each item was graded as low, high, or unclear. Two reviewers assessed the risk of bias independently, with any discrepancies resolved through consensus discussions or the additional judgment of a third reviewer.

### 2.7 Certainty of evidence assessment

The CINeMA (Confidence in Network Meta-Analysis) online tools were used to evaluate the confidence in the synthesized results. CINeMA includes six domains that affect the level of confidence in the network meta-analysis results: (1) within-study bias, (2) reporting bias, (3) indirectness, (4) imprecision, (5) heterogeneity, and (6) incoherence. Each network meta-analysis result is rated on a three-level scale: “no concerns”, “some concerns”, or “major concerns” in terms of each of the six domains. Finally, judgments across the domains were summarized into a single confidence rating of “high”, “moderate”, “low”, or “very low”.

### 2.8 Statistical analysis

To generate more comprehensive and precise results, we performed not only traditional paired meta-analyses on direct data but also network meta-analyses on both direct and indirect data. This allowed us to synthesize the effect sizes of different interventions and to compare the differences between them. Changes in different outcome variables were analyzed using data from the fourth-week post-intervention follow-up as the primary endpoint; when fourth-week post-intervention data were unavailable, the closest available time points were utilized for analysis. RevMan 5.3, Stata 16, and ADDIS 1.16.5 were used for statistical analysis. Statistically significant differences were set at *P* < 0.05.

(1) Pairwise meta-analysis.

The standardized mean difference (SMD) and its 95% confidence interval (95% CI) were calculated for each outcome variable. The overall effect size was computed using Z-statistics with a significance level of *P* < 0.05.

Statistical heterogeneity was assessed using the standard chi-squared and *I^2^* statistics (24). Heterogeneity was interpreted as not important (≤ 40%), moderate (30−60%), substantial (50−90%), or considerable (≥ 75%) (22). Overall, associations between different interventions and perinatal psychological outcomes were analyzed using a random effects model if I^2^ > 50%, while fixed effects models were applied if I^2^ ≤ 50.

When substantial heterogeneity was identified, a multivariate meta-regression analysis was conducted to investigate the potential sources of heterogeneity, including publication year, geographic region, intervention platform (online vs. face-to-face), purpose (prevention vs. treatment), and guidance approach (self-help vs. therapist-assisted). Moreover, sensitivity analyses were performed by excluding studies with a high risk of bias to assess their impact on the overall estimates. In addition, publication bias was visually assessed by the symmetry of a funnel plot and, when applicable, statistically evaluated using Egger’s test (25).

(2) Network meta-analysis.

Bayesian network meta-analyses were conducted using ADDIS (Automated Data and Document Integration System), version V1.16.5, a powerful, open-source software platform that supports evidence-based decision-making in healthcare. This analysis runs Markov chain Monte Carlo simulations with four chains, 20,000 tuning iterations, 50,000 simulation iterations, a thinning interval of 10, 10,000 inference samples, and a variance scaling factor of 2.5. Model convergence was assessed using the Brooks-Gelman-Rubin diagnostic statistics. Since the outcome indicators were continuous variables, the mean difference with 95% CIs was used to demonstrate the results.

Network diagrams were also used to visualize the direct and indirect comparisons across different interventions. In these network diagrams, the size of an intervention node represents the sample size of the intervention population, and the thickness of the line reflects the number of included studies. If a closed loop occurs, an inconsistency analysis is adopted using loop-specific and side-splitting methods to assess the difference between direct and indirect comparisons.

Moreover, a surface under the cumulative ranking (SUCRA) curve analysis was conducted to estimate the ranking probabilities of the intervention efficacy among different interventions. The hierarchy ranking of each intervention approach was revealed as a P-score and a SUCRA cumulative surface area map. The P-score, derived from SUCRA, represents the probability that a treatment is the best of all the interventions compared in the network meta-analysis. It ranges from 0 to 1, with higher values indicating a greater likelihood of being the best option. The SUCRA cumulative surface area map uses SUCRA values to aggregate the probabilities of each intervention’s ranking and aggregates the cumulative probability results for all interventions. Regarding publication bias, although some individual comparisons included fewer than 10 studies, the funnel plot and Egger’s test were still performed, given the large number of studies included in the overall analysis.

## 3 Results

### 3.1 Study selection

**Figure 1:**
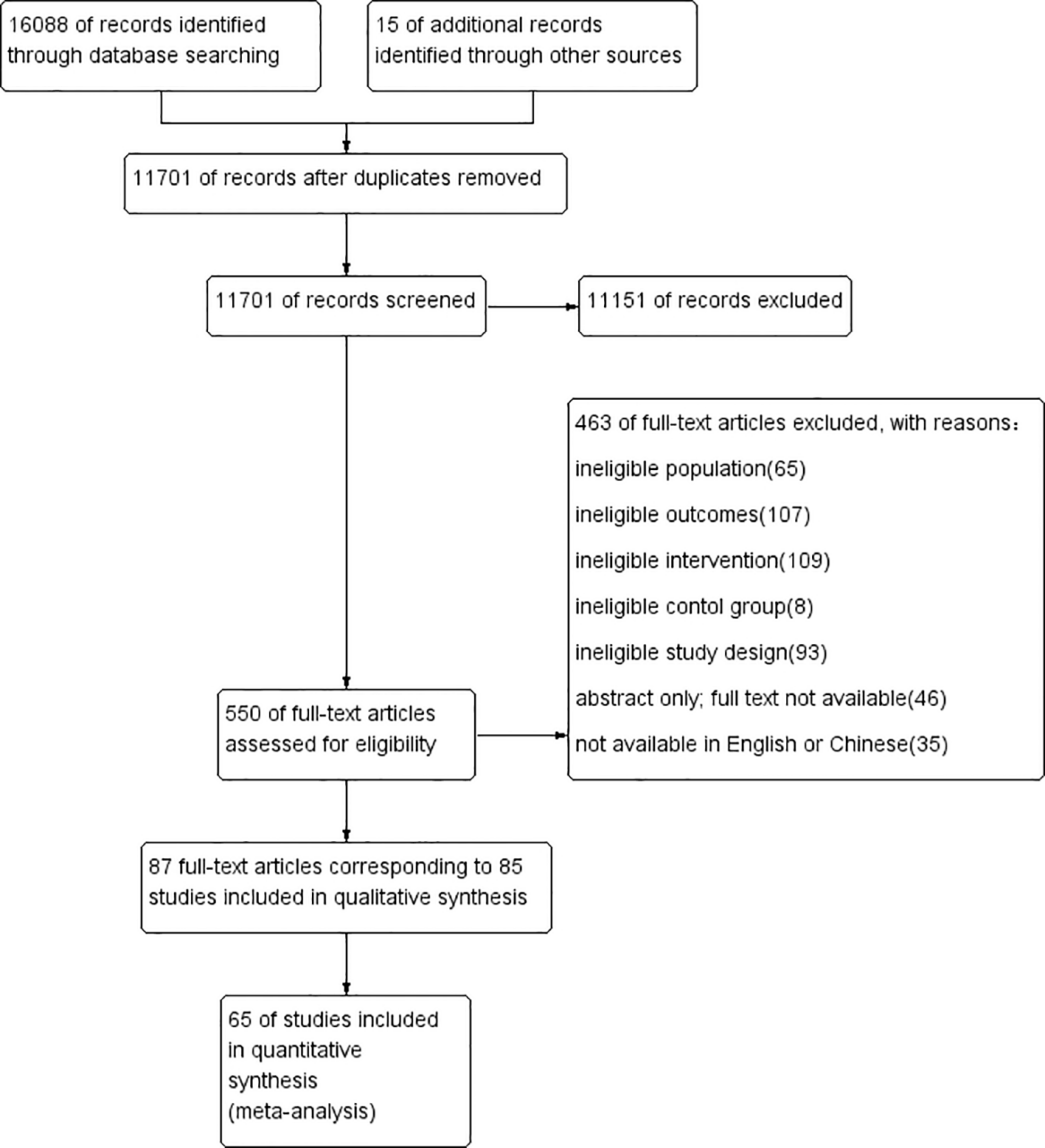
Flow diagram of study selection.

As illustrated in **Figure 1**, 16,088 records were identified through database searching, with an additional 15 records captured via manual search. After removing duplicates, 11,701 publications remained. Of those, 11,151 were excluded based on title and abstract screening. The remaining 550 publications were further assessed for eligibility at the full-text level. Finally, 87 publications reporting 85 studies were included in the qualitative synthesis, and 65 of these were included in the meta-analysis.

### 3.2 Characteristics of the included studies

**Table 1** and **Supplementary Table 2** show the general characteristics and details of the interventions of the 85 trials, which involved 19,608 pregnant women in total, with a mean age ranging from 20.1 to 36.8 years. Of the included studies, one was three-armed, and the rest were two-armed. Fifty-eight studies (68.2%) recruited participants from Asia, fourteen (16.5%) from Europe, eight (9.4%) from the Americas, three (3.5%) from Oceania, and two (2.4%) from Africa. Eight types of psychotherapies and psychosocial interventions were examined, including cognitive behavioral therapy (n=31), mindfulness-based interventions (n=19), acceptance and commitment therapy (n=1), interpersonal therapy (n=5), psychoeducation (n=9), counseling (n=5), multicomponent interventions (n=14), and behavioral interventions (n=1). In the majority of the studies (n=73), the interveners were professional psychological therapists. Regarding the interventions’ aims, 76 were for prevention and six were for treatment, with the remaining three unspecified.

**Table 1:** General characteristics of the included studies.

Primary author, publication year country/region	Participants’ gestation week	Age (Mean±SD)		Sample size		Intervention components	Outcomes (Measurement)	ITT/MDM
		IG	CG	IG	CG			
Abdollahi, 2020 Iran	26-33	25.5±5.3	24.5±4.3	35	35	Counseling (Motivational interviewing)	Anxiety (Spielberger state-anxiety questionnaire); Stress (NuPDQ)	N/N
Abujilban, 2024 Jordan	24-37	28.8	29.2	50	50	IPT	Depression (EPDS)	Y/N
Alipour, 2020 Iran	<24	29.1±4.3	29.4±4.5	30	30	Psychoeducation	Depression & Anxiety (GHQ)	N/N
Baniaghil, 2022 Iran	12-20	26.2±4.6	25.5±4.4	53	61	MBI	Stress (PWSQ)	N/N
Bayat, 2021 Iran	11-15	28.8±5.4	28.2±6.7	46	46	CBT	Anxiety (STAI)	Y/Y
Bittner, 2014 Germany	10-15	29.4±3.6	29.7±4.7	80	80	CBT	Depression (EPDS); Anxiety (STAI)	N/Y
Chan, 2019 Hong Kong	<24	31.3±4.6	31.2±4.5	330	330	Psychoeducation	Depression (EPDS); Anxiety & Stress (DASS)	Y/Y
Cui, 2023 China	24-32	32.7±4.6	32.0±4.3	37	37	CBT	Depression (EPDS); Anxiety (PAQ); Stress (PSS-10)	N/N
Esfandiari, 2020 Iran	6-32	27.9±5.3	23.7±4.3	40	40	Supportive counseling	State anxiety (Spielberger state-anxiety questionnaire); Perceived stress (PSS-14), Pregnancy-related stress (NuPDQ)	Y/Y
Eteraf, 2023 Iran	6-32	29.1±6.1	29.7±6.6	50	50	Supportive counseling	Stress (CSS-18)	N/N
Felder, 2020 USA	≤28	33.6±3.7		105	103	CBT	Depression (EPDS); Anxiety (GAD-7)	Y/N
Forsell, 2017 Sweden	12-28	31.2±3.7	30.8±5.3	22	20	CBT	Depression (MADRS-S & EPDS); Anxiety (GAD-7)	Y/Y
Gao, 2012 China	≥28	28.5±2.8	28.4±2.7	96	98	IPT	Depression (EPDS);	Y/N
Gedde-Dahl, 2012 Norway	3^rd^ trimester	30.6±4.1	30.5±3.6	29	29	BI (Self-administered relaxation and guided imagery)	Depression (BDI); Anxiety (STAI-S&T);	N/N
Golshani, 2021 Iran	14-20	31.8±5.9	31.1±5.3	28	28	CBT	Depression (EPDS); Anxiety (PRAQ); Stress (PSS)	Y/N
Grote, 2009 USA	10-32	24.3±5.3	24.7±5.6	25	28	IPT	Depression (EPDS & BDI); Anxiety (BAI)	Y/Y
Hassdenteufel, 2023 Germany	<29	32.3±4.7	32.8±4.6	230	230	MBI	Depression (EPDS); State Anxiety (STAI); Pregnancy-related anxiety (PRAQ-R); Stress (PHQ-D stress)	N/Y
Heller, 2022 Netherlands	NR	32.1±4.6	31.9±4.8	79	80	Multicomponent interventions (PST & Psychoeducation)	Depression (CES-D, EPDS); Anxiety (HADS-A)	Y/Y
Huang, 2015 China	36	27.3±2.7	25.8±3.1	120	120	CBT	Depression (SDS); Anxiety (SAS); Stress (PSS)	NR/NR
Hulsbosch, 2023 Netherlands	<12	31.2±3.5	31.2±4.0	109	110	MBI	Depression (EDS); Anxiety (TPDS-NA)	Y/Y
Jesse, 2015 USA	6-30	24.9±5.6	25.2±5.4	72	74	CBT	Depression (EPDS & BDI-II);	N/N
(1) Kaboli, 2017 Iran (2) Salehi, 2020 Iran	20-32	NR	NR	35	35	CBT	Stress (PWSQ)	N/N
Kalmbach, 2020 USA	25-30	29.0±4.2		46	45	CBT	Depression (EPDS)	Y/Y
Khamseh, 2019 Iran	NR	27.8±4.6	26.3±4.0	35	35	Multicomponent interventions (PST & Psychoeducation)	Depression (BDI)	NA/NA
Kharaghani, 2023 Iran	<14	34.3±6.6	32.2±5.9	21	21	CBT	Anxiety (PRAQ); Stress (CWS)	NA/NA
Khatibi, 2021 Iran	12-24	27.3±5.4	25.6±4.5	28	28	CBT	Depression & Anxiety & Stress (DASS-21)	N/N
Khorsandi, 2015 Iran	18-32	20.1±1.4	20.4±1.9	32	32	CBT	Perceived stress (PSS)	N/N
Kozinszky, 2012 Hungary	<25	27.3±4.2	27.4±3.9	728	1034	Multicomponent interventions (psychoeducation & IPT & CBT)	Depression (LQ)	Y/N
Kuo, 2022 Taiwan	12-24	34±4	33.7±4.8	56	56	Multicomponent interventions (Mindfulness practice & childbirth education)	Depression (EPDS); Anxiety (STAI)	N/Y
Le, 2011 USA	≤24	25.8±4.4	25.0±4.8	112	105	CBT	Depression (BDI-II)	Y/Y
Leung, 2012 HongKong	12-32	31.3±4.0	31.2±4.1	78	78	IPT	Depression (EPDS); Stress (PSS)	Y/Y
Li, 2014 China	12-18	26.8±6.2		70	78	Multicomponent interventions (CBT & biofeedback therapy)	Depression (HAMD); Anxiety (HAMA)	NR/NR
Li, 2020 China	6-8	28.5±3.6	28.2±3.2	50	50	CBT(CBSM)	Stress (PPS)	NA/NA
Li, 2021 China	≤28	26.3±3.2	26.2±2.9	45	45	Multicomponent interventions (Psychoeducation & situation simulation training)	Depression (EPDS); Anxiety (SAS)	NR/NR
Li, 2022 China	≥28	26.9±5.9	27.5±3.8	42	42	Multicomponent interventions (Psychoeducation &peer support)	Depression (SDS); Anxiety (HAMA)	NR/NR
Li, 2023 China	NR	27.8±5.7	28.6±5.5	30	30	Multicomponent interventions (CBT & Mindfulness)	Depression (SDS); Anxiety (SAS)	NR/NR
Liao, 2014 China	≥36	28.7±4.3	29.1±5.0	150	150	CBT	Depression (SDS); Anxiety (SAS)	NR/NR
Lönnberg, 2021 Sweden	15-22	32±3.9	32±4.1	96	97	MBI	Depression (EPDS); Stress (PSS)	Y/Y
Loughnan, 2018 Australia	13-30	31.6±4.0		43	44	CBT	Depression (PHQ-9 & EPDS & BDI-II); Anxiety (GAD-7)	N/Y
Lowndes, 2018 Australia	≥28	NR	NR	30	30	CBT	Depression (EPDS)	Y/N
Lund, 2020 South Africa	≤28	NR	NR	216	209	Multicomponent interventions (Psychological counseling & psychoeducation)	Depression (HDRS & EPDS)	Y/Y
MacKinno, 2021 Canada	12-28	30.4±5.3	32.9±4.4	28	32	MBI (mindfulness-based cognitive therapy)	Depression (EPDS); Anxiety (GAD-7); Anxiety (PRA); Stress (PSS)	N/Y
Mahmoudi, 2023 Iran	20-32	30.2±5.7	31.1±5.8	38	38	Psychoeducation	Depression (EPDS); Anxiety (Spielberger state-trait anxiety); Stress (PDQ)	N/Y
Mao, 2012 China	32	28.5±2.4	28.8±2.5	120	120	CBT	Depression (PHQ-9 & EPDS)	N/N
Mao, 2021 China	0-12	27.1±6.4	28.1±6.7	35	35	Multicomponent interventions (Psychoeducation & peer support)	Depression (SDS); Anxiety (SAS)	Y/N
Mckee, 2006 China	≤32	24.6±5.6	24.2±5.6	57	43	Multicomponent interventions (CBT&health education & Social support)	Depression (BDI-II)	NR/NR
Mei, 2023 China	≤28	26.7±5.2	28.0±5.2	30	30	CBT	Anxiety (HARS)	Y/N
Missler, 2020 Netherlands	26-34	32.7±3.4	32.2±3.5	68	69	Psychoeducation	Depression (EPDS); Anxiety (HADS-A); Parenting stress (PSI)	N/N
Mokaberian, 2021 Iran	28-30	NR	NR	30	30	Multicomponent interventions (muscle relaxation & imagery-based relaxation & psychoeducation)	Depression & Anxiety & Stress (DASS-21)	Y/Y
Montazeri, 2020 Iran	28-31	27.5±5.9	27.7±5.8	35	35	Writing therapy-based counseling	Anxiety (BAI)	NA/NA
Mortazavi, 2021 Iran	28-32	26.2±5.3	26.1±4.9	66	66	Solution-focused counseling	Anxiety (ASP)	N/N
Mu, 2014 China	NR	26.3±4.1	25.3±3.2	37	37	CBT	Depression (SDS); Anxiety (SAS)	N/N
Muthukrishnan, 2016 India	12	21±2.6	23±2.4	37	37	MBI	Stress (PSS)	NR/NR
Nazari, 2018 Iran	14-25	28.8±4.9	27.0±5.2	50	50	Fordyce Happiness CBT	Stress (PSS-14)	NR/NR
Nejad, 2021 Iran	<32	28.9±5.6	29.3±6.3	30	30	MBI (mindfulness-based stress reduction)	Depression & Anxiety & Stress (DSAA-21)	NA/NA
Ngai, 2022 Hongkong	12-30	33.3±3.5	32.1±3.9	224	231	IPT	Depression (EPDS)	Y/ N
Nishi, 2022 Japan	16-20	30.4±4.6	30.5±4.6	2509	2508	CBT	Depression (EPDS)	Y/ N
Pan, 2019 Taiwan^a, b^	13-28	32.7±3.8	33.0±3.9	52	52	MBI	Depression (EPDS); Stress (PSS-10)	Y/N
Pan, 2023 Taiwan	13-28	33.5±4.9	32.9±3.9	51	51	MBI	Depression (EPDS); Stress (PSS-10)	N/N
Pinar, 2017 Turkey	8-30	27.4±4.4	27.9±5.1	110	110	Psychoeducation	Depression (BDI), Stress (PSS)	N/N
(1) Ponting, 2022 USA (2) Urizar, 2019 USA	<17	26.3±6.7	26.8±6.0	55	45	CBT(CBSM)	State anxiety (STPI-S), pregnancy-specific anxiety (PRA); Stress (PSS-14)	N/N
Puertas-Gonzalez, 2022 Spain	12-28	IG1:35.1±3.6 IG2:35.6±4.4	34.3±4.9	IG1:70 IG2:69	68	IG1: CBT IG2: Psychoeducation	Depression & Anxiety (SCL-90-R); Stress (PSS-14); Pregnancy-specific stress (PDQ)	Y/ Y
Ren, 2021 China	>12	30 (28, 32)^#^	29 (28, 31)^#^	85	85	CBT	Depression (EPDS); Anxiety (PAQ)	Y/NR
Richter, 2012 Germany	10-15	29.2±4.5	30.0±4.3	80	81	CBT	Stress (PDQ); perceived stress (PSS)	Y/NR
Romero-Gonzalez, 2020 Spain	12-28	34±5.0	32.0±4.0	46	47	CBT	Stress (PDQ), Perceived stress (PSS)	N/N
Rouhe, 2015 Finland	11-13	NR	NR	131	240	Psychoeducation	Depression (EPDS)	Y/N
Sanaeinasab, 2020 Iran	1^st^ trimester	26.4±2.9		42	42	CBT	Depression& Anxiety & Stress (DASS)	N/Y
Scherer, 2016 Switzerland	18-32	32.9±3.49	32.1±3.5	51	42	CBT	Anxiety (STAI-S); Stress (PSS)	N/N
Shamabadi, 2023 Iran	NR	28.1±5.4	28.1±5.9	50	48	MBI	Depression & Anxiety & Stress (DASS-21)	N/N
Smith, 2021 USA	14-34	35.6±9.9	36.8±12.6	50	51	MBI	Depression & Anxiety (HADS); Stress (PSS)	NR/NR
Sun, 2021 China	12-20	30.3±3.8	29.6±4.2	84	84	MBI	Depression (EPDS & PHQ-9); Anxiety (GAD-7); Stress (PSS)	Y/ N
Surkan, 2023 Pakistan	≤22	25.1±4.6	25.5±4.7	600	600	CBT	Depression (HADS-D & PHQ-9); Anxiety (HADS-A)	Y/Y
Tessema, 2024 Ethiopia	12-20	NR	NR	286	264	Psychoeducation	Depression (PHQ-9)	Y/N
Toohill, 2014 Australia	≤24	29±5.9	29.2±5.0	170	169	Psychoeducation	Depression (EPDS)	Y/N
Vakilian, 2019 Iran	NR	22.6±1.5	22.1±1.4	22	22	ACT	Anxiety (PRAQ)	NA/NA
Wu, 2023 China	≥28	NR	NR	45	45	CBT	Anxiety (PAQ)	Y/NA
Yan, 2024 China	28	25.9±2.5	25.8±2.7	91	91	Multicomponent interventions (Psychoeducation &situation simulation training)	Depression (SDS); Anxiety (SAS)	N/N
Yang, 2019 China	24-30	31.3±5.0	30.4±3.9	62	61	MBI	Depression (PHQ-9); Anxiety (GAD-7)	NR/NR
Yazdanimehr, 2016 Iran	1-6	26±5.8	26.7±4.5	40	40	Multicomponent interventions (MBI &CBT)	Depression (EPDS); Anxiety (BAI)	Y/Y
Zareneiad, 2020 Iran	24-36	27±5	24.5±5	35	35	MBI (mindfulness-based stress reduction)	Anxiety (PRAQ)	N/N
Zemestani, 2019 Iran	1-6	28.6±3.0	30.5±4.2	19	19	MBI (mindfulness-based cognitive therapy)	Depression (BDI-II); Anxiety (BAI)	N/N
Zhang, 2018 China	14-28	25.7±2.8	25.6±2.3	34	32	MBI (mindfulness-based stress reduction)	Depression (SDS); Anxiety (STAI); Stress (PSRS)	Y/Y
Zhang, 2022 China	12-20	30.4±5.0	30.1±3.9	80	80	MBI	Depression (EPDS); Anxiety (GAD-7)	N/N
Zhang, 2023 China	12-20	30.4±4.7	30.2±3.9	80	80	MBI	Depression (EPDS); Anxiety (GAD-7); Pregnancy-related anxiety(PRAS)	N/N
Zhang, 2023 China	12-24	28.5±3.7	2.92±3.5	54	54	MBI	Depression (EPDS); Anxiety (GAD-7); Stress (PSS-4)	Y/Y

ITT, Intention-to-treat analysis; MDD, Missing data management; IG, intervention group; CG, control group; MBI, mindfulness-based intervention; CBT, cognitive behavioral therapy; CBSM, cognitive behavioral stress management; IPT, interpersonal therapy; ACT, acceptance and commitment therapy; PST, problem-solving therapy; BI, behavioral intervention; TAU, treatment as usual; EPDS, Edinburgh Postnatal Depression Scale; PSS, Perceived Stress Scale; HADS, Hospital Anxiety and Depression Scale; PHQ, Patient Health Questionnaire; SAS, Self-rating Anxiety Scale; CES-D, Center for Epidemiological Studies Depression Scale; LQ, Leverton Questionnaire; PSI , Parenting Stress Index; PPS, Pregnancy Pressure Scale; STPI, State-Trait Personality Inventory; DASS, Depression, Anxiety, and Stress Scale; CAQ, Childbirth Attitude Questionnaire; STAI, State-Trait Anxiety Inventory; PWSQ, Pregnancy Worries and Stress Questionnaire; PRAQ, Pregnancy-related Anxiety Questionnaire; SAI, State Anxiety Inventory; SCL, Symptom Checklist; PDQ, The Prenatal Distress Questionnaire; NuPDQ, the revised Prenatal Distress Questionnaire; BAI, Beck Anxiety Inventory; ASP, Anxiety Scale for Pregnancy; GAD, Generalized Anxiety Disorder; CSS, the Corona Stress Scale Questionnaire; MADRS, Montgomery-Asberg Depression Rating Scale Self-report version; GHQ, General Health Questionnaire; TPDS-NA, the Tilburg Pregnancy Distress Scale-negative affect; BDI, Beck Depression Inventory; K-6, Kessler’s Psychological Distress Scale; CWS, Cambridge Worry Scale; PRA, Pregnancy-related Anxiety Scale; SDS, Self-rating Depression Scale; HAMA, Hamilton Anxiety Scale; NR, Not reported; NA, Not applicable; ^#^: The non-normal distribution is denoted by M (P25, P75).

### 3.3 Methodological quality of the included studies

**Figure 2:**
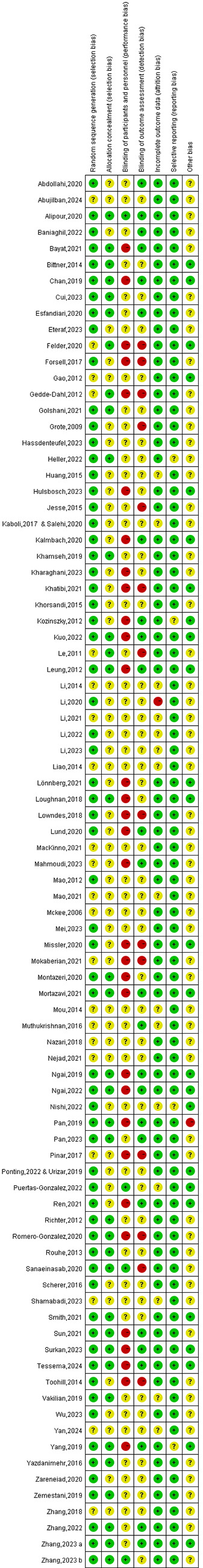
Risk of bias of individual studies.

**Figure 3:**
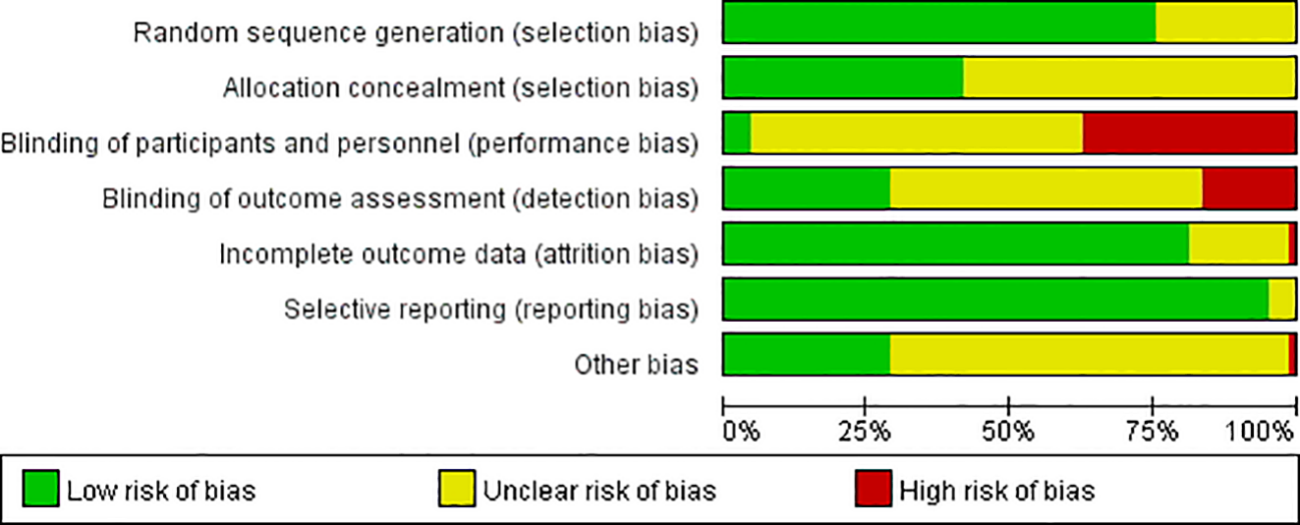
Risk of bias summary of included studies.

The risk of bias for the included studies is presented in **Figures 2** and **3**. Of the 85 included trials, 32 (37.6%) were rated as low risk, 39 (45.9%) as moderate risk, and the remaining 14 (16.5%) as high risk. While all studies mentioned randomizing participants, many failed to report the specific methods or provide details on allocation concealment, raising concerns about selection bias. Approximately one-third of the studies did not blind the research participants and/or interveners, while 16.5% failed to blind the outcome assessors. The majority of the studies had low-risk concerns in reporting bias; however, only approximately one-third of them were of low risk in other biases.

### 3.4 Results on the effectiveness of prenatal psychotherapies and psychosocial interventions

#### 3.4.1 Symptoms of depression

**Figure 4:**
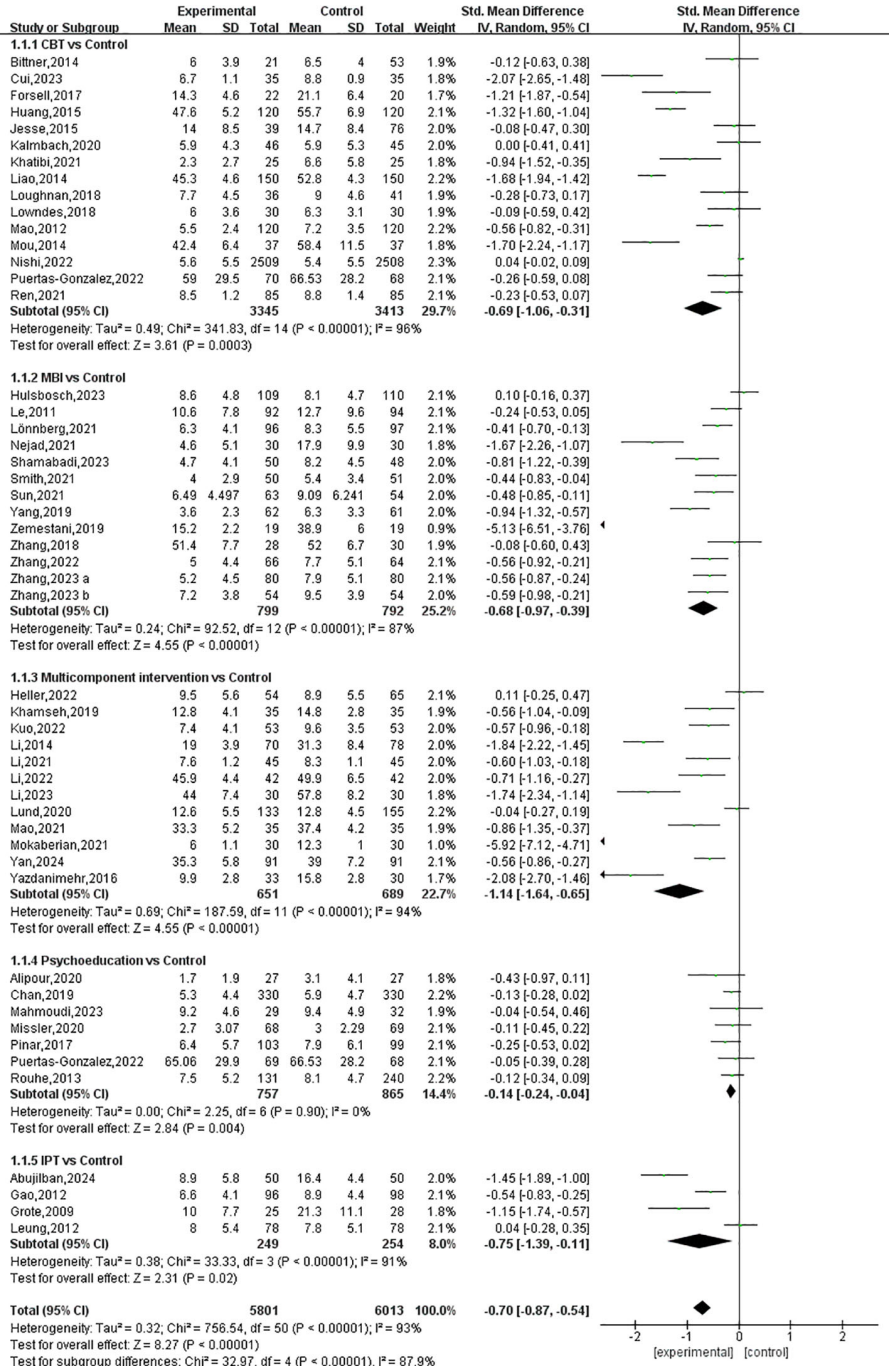
.The Forest plot of the effects of prenatal psychotherapies and psychosocial interventions on depressive symptoms.

Symptoms of depression were assessed in 50 of the included studies, involving 5,801 pregnant women in the experimental groups and 6,014 in the control groups. As shown in **Figure 4**, the synthesized results demonstrate a significant beneficial effect of prenatal psychotherapies and psychosocial interventions on symptoms of depression, with an SMD of -0.70 (95% CI: -0.87 to -0.54).

**Figure 5:**
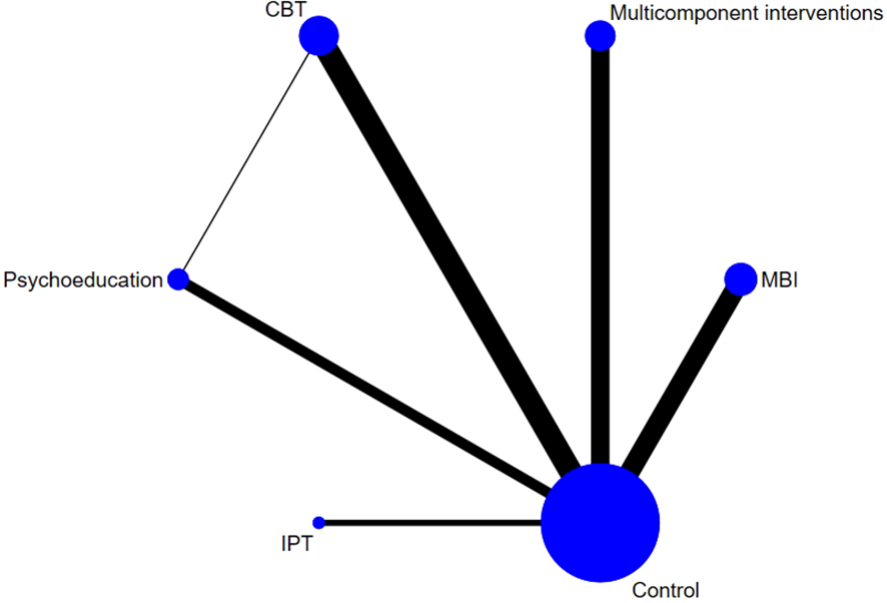
Network diagrams for depressive symptoms.

All of the five examined interventions were more effective than the control interventions; however, the effect sizes differed: multicomponent interventions, IPT, CBT, MBIs, and psychoeducation were effective in significantly alleviating symptoms of depression by SMDs of -1.14 (95% CI: -1.64 to -0.65), -0.75 (95% CI: -1.39 to -0.11), -0.69 (95% CI: -1.06 to -0.31), -0.68 (95% CI: -0.97 to -0.39) and -0.14 (95% CI: -0.24 to -0.04), respectively, compared to control interventions (**Figures 4**, **5**). The P-score derived from the SUCRA curve is the highest for multicomponent interventions (0.934), followed by IPT (0.639), MBIs (0.621), CBT (0.571), and psychoeducation (0.191), also suggesting that multicomponent interventions could be a preferred option for managing perinatal symptoms of depression (**Supplementary Figure 1**, **Supplementary Table 3**).

Between-group comparisons across the five interventions showed that multicomponent interventions may produce better effects compared to psychoeducation (SMD=-0.90, 95% CI: -1.50 to -0.30). No significant differences were detected between the other interventions (**Table 2**).

**Table 2:** Relative effects of the different prenatal psychotherapies and psychosocial interventions for depressive symptoms (Random model).

Interventions Comparators	Multicomponent interventions	MBI	CBT	Psychoeducation	IPT
MBI	-0.36 (-0.88, 0.16)				
CBT	-0.41 (-0.91, 0.09)	-0.05 (-0.54, 0.44)			
Psychoeducation	-0.90 (-1.50, -0.30)*	-0.54 (-1.13, 0.05)	-0.49 (-1.04, 0.06)		
IPT	-0.34 (-1.08, 0.41)	0.02 (-0.71, 0.76)	0.07 (-0.65, 0.79)	0.56 (-0.23, 1.36)	
Control	-1.10 (-1.48, -0.72)*	-0.74 (-1.10, -0.38)*	-0.69 (-1.01, -0.36)*	-0.20 (-0.66, 0.27)	-0.76 (-1.40, -0.12)*

*P<0.05; MBI, Mindfulness-based intervention; CBT, Cognitive behavioral therapy; IPT, Interpersonal treatment; ACT, Acceptance and commitment therapy.

#### 3.4.2 Symptoms of anxiety

**Figure 6:**
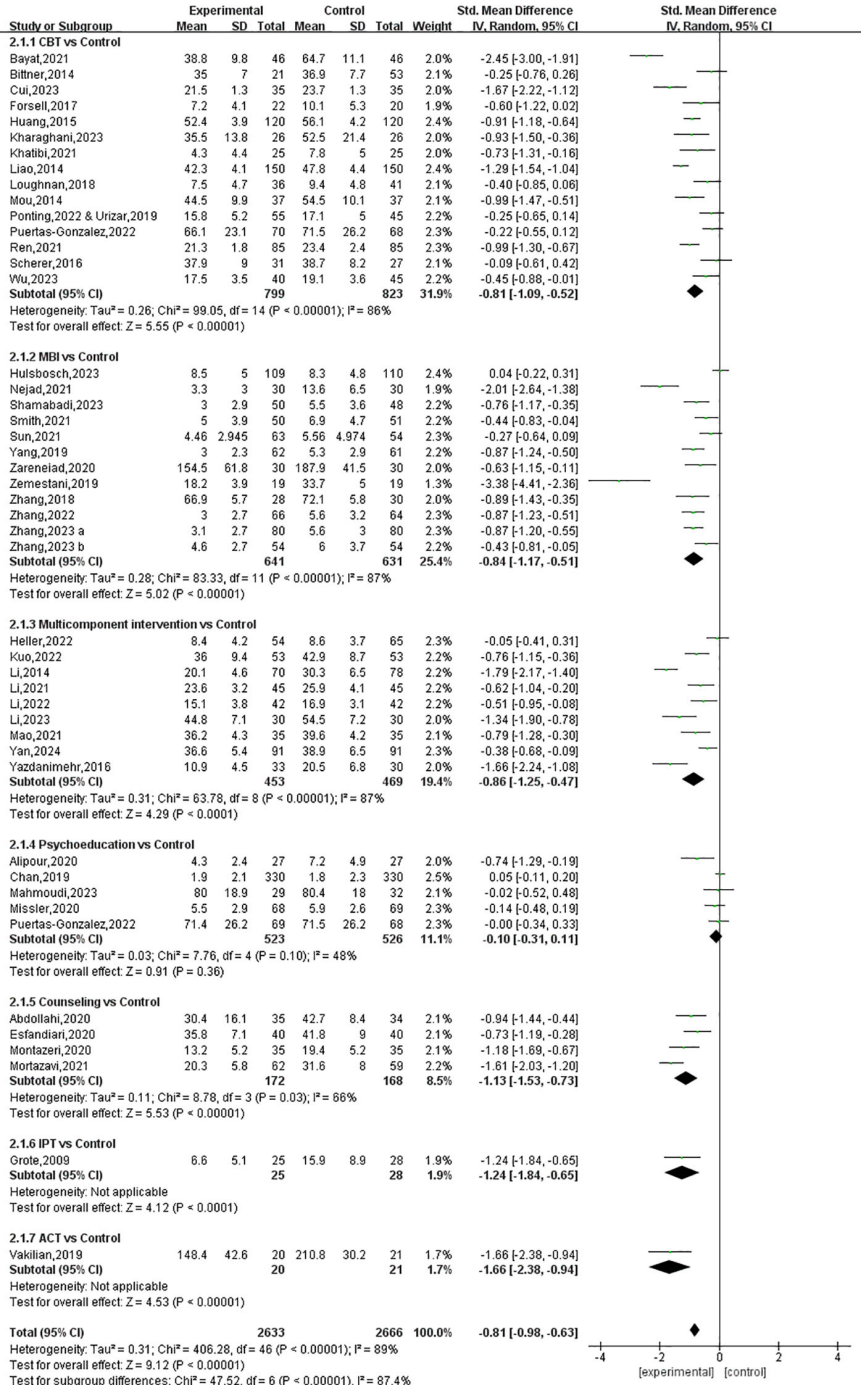
The Forest plot of the effects of prenatal psychotherapies and psychosocial interventions on anxious symptoms.

Among the 47 studies that measured symptoms of anxiety, one presented incorrect data and was therefore excluded from the analysis. In the remaining studies, conducted among a total of 5,231 pregnant women, the effectiveness of seven types of prenatal psychotherapies and psychosocial interventions was investigated. As is illustrated in **Figure 6**, the synthesized results demonstrate a significant beneficial effect of prenatal psychotherapies and psychosocial interventions on symptoms of anxiety, with an SMD of -0.81 (95% CI: -0.98 to -0.63).

**Figure 7:**
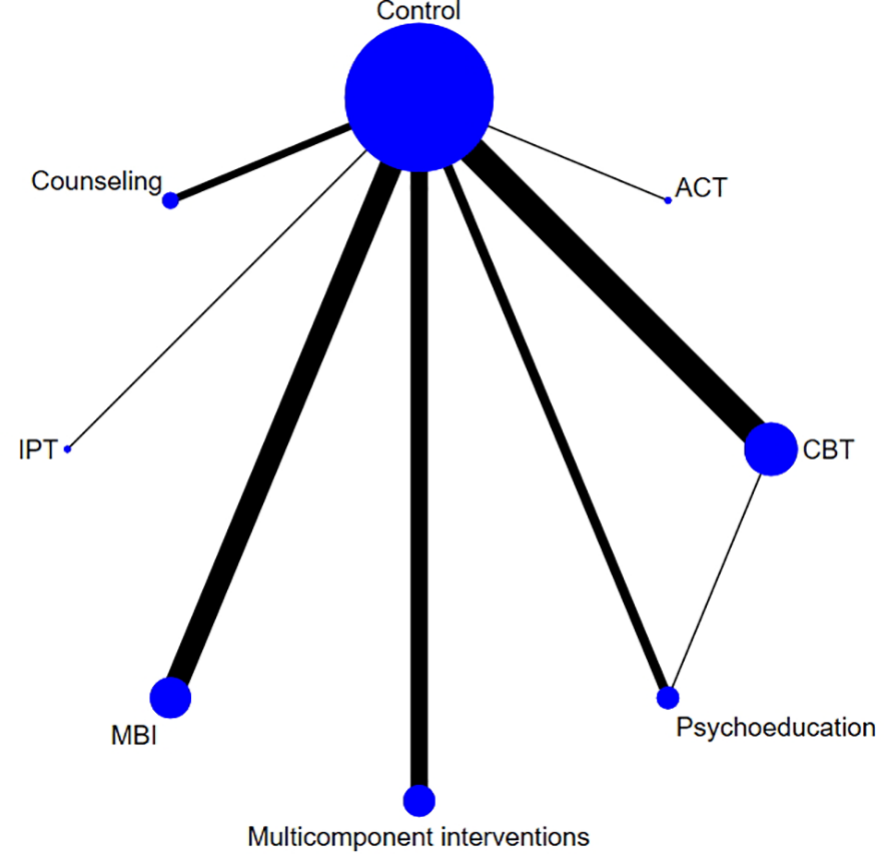
Network diagrams for anxious symptoms.

All interventions except psychoeducation (SMD: -0.10, 95% CI: -0.31 to 0.11) were found to be effective in relieving symptoms of anxiety. The effect size was largest with ACT (SMD: -1.66, 95% CI: -2.38 to -0.94), followed by IPT (SMD: -1.24, 95% CI: -1.84 to -0.65), counseling (SMD: -1.13, 95% CI: -1.53 to -0.73), multicomponent intervention (SMD: -0.86, 95% CI: -1.25 to -0.47), MBIs (SMD: -0.84, 95% CI: -1.17 to -0.51) and CBT (SMD: -0.81, 95% CI: -1.09 to -0.52) (**Figures 6**, **7**). The P-score derived from the SUCRA is the highest for ACT (0.875), followed by counseling (0.739), IPT (0.731), MBIs (0.590), CBT (0.549), and multicomponent interventions (0.310), also suggesting that ACT could be a superior option for managing perinatal symptoms of anxiety (**Supplementary Figure 2**, **Supplementary Table 3**). However, it is worth noting that only one study on ACT and one study on IPT were included in the analysis; therefore, the reported effect sizes were subject to a low level of generalizability.

Between-group comparisons across the seven interventions showed that multicomponent interventions and counseling could produce greater effects compared to psychoeducation (SMD=-0.64, 95% CI: -1.21 to -0.07 and SMD=-0.91, 95% CI: -1.61 to -0.21, respectively) (**Table 3**).

**Table 3:** Relative effects of the different prenatal psychotherapies and psychosocial interventions for anxious symptoms (Random model).

Multicomponent interventions							
-0.02 (-0.49, 0.45)	MBI						
-0.05 (-0.50, 0.39)	-0.03 (-0.44, 0.38)	CBT					
-0.64 (-1.21, -0.07)*	-0.62 (-1.16, 0.07)	-0.59 (-1.10, 0.08)	Psychoeducation				
0.27 (-0.37, 0.91)	0.29 (-0.33, 0.91)	0.32 (-0.28, 0.92)	0.91 (0.21, 1.61)*	Counseling			
0.40 (-0.78, 1.58)	0.42 (-0.75, 1.59)	0.45 (-0.71, 1.61)	1.04 (-0.18, 2.26)	0.13 (-1.12, 1.38)	IPT		
-0.83 (-0.42, 2.08)	0.86 (-0.38, 2.09)	0.89 (-0.34, 2.11)	1.47 (-0.19, 2.75)	0.56 (-0.75, 1.88)	0.43 (-1.21, 2.08)	ACT	
-0.86 (-1.22, -0.51)*	-0.84 (-1.15, -0.53)*	-0.81 (-1.08, -0.54)*	-0.22 (-0.67, 0.23)	-1.13 (-1.67, -0.60)*	-1.26 (-2.39, 0.13)	-1.70 (-2.90, -0.50)*	Control

*P<0.05; MBI, Mindfulness-based intervention; CBT, Cognitive behavioral therapy; IPT, Interpersonal treatment; ACT, Acceptance and commitment therapy.

#### 3.4.3 Stress

**Figure 8:**
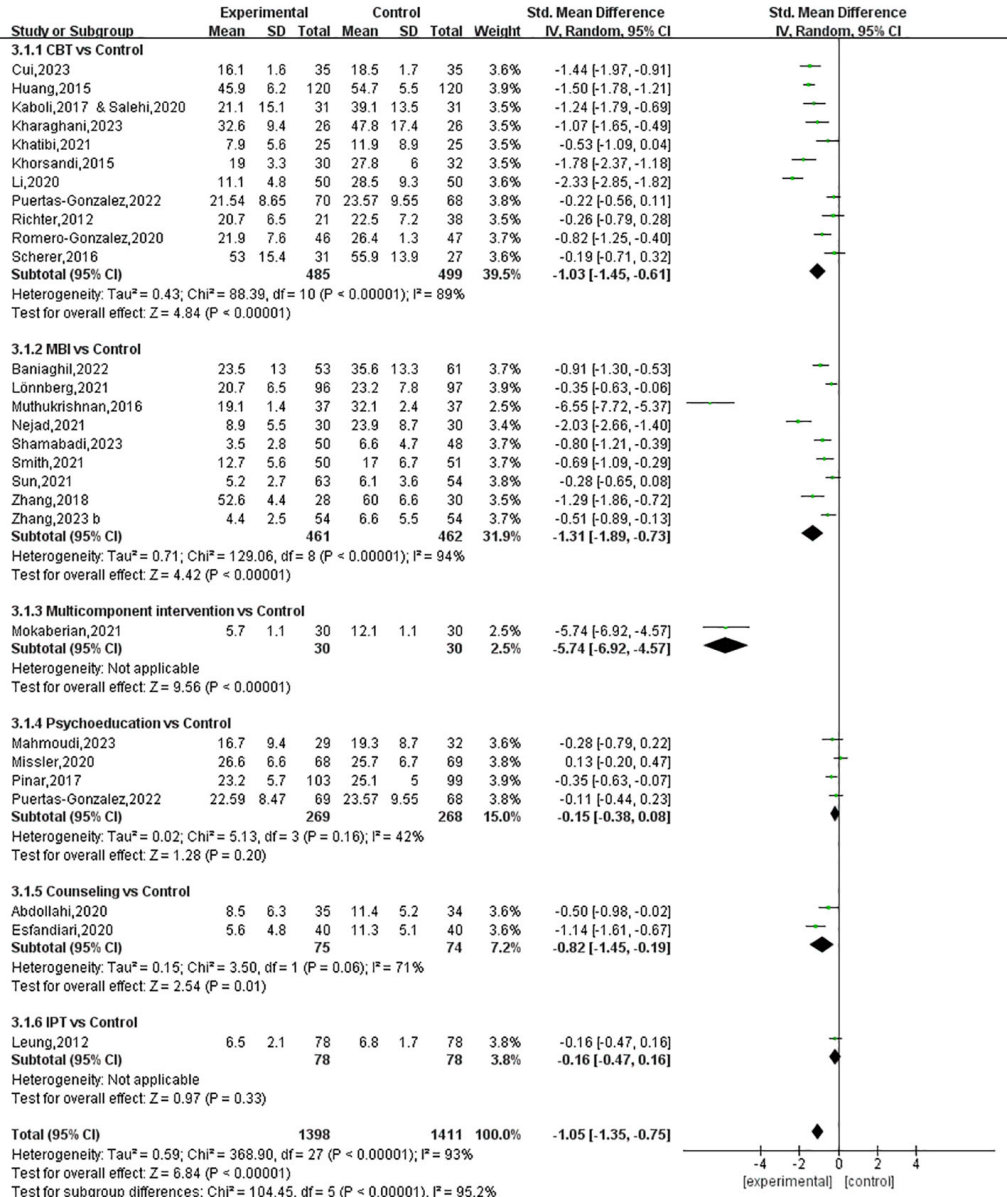
The Forest plot of the effects of prenatal psychotherapies and psychosocial interventions on stress.

Stress was measured in 27 studies involving 2,741 participants. The pooled results of six types of prenatal psychotherapies and psychosocial interventions show favorable effects in alleviating stress symptoms, with an SMD of -1.05 (95% CI: -1.35 to -0.75) (**Figure 8**).

**Figure 9:**
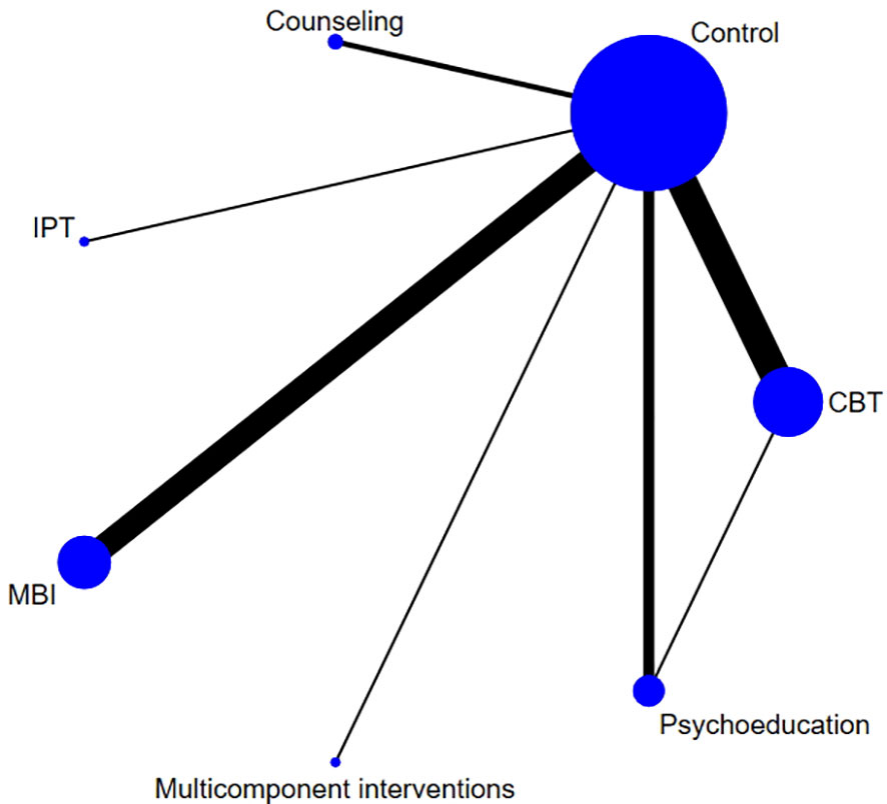
Network diagrams for stress.

Among the six types of interventions, multicomponent interventions (SMD: -5.74, 95% CI: -6.92 to -4.57), MBIs (SMD: -1.31, 95% CI: -1.89 to -0.73), CBT (SMD: -1.03, 95% CI: -1.45 to -0.61) and counseling (SMD: -0.82, 95% CI: -1.45 to -0.19) were effective strategies for the management of perinatal stress, while psychoeducation and IPT were found to be ineffective (**Figures 8**, **9**). The P-score derived from the SUCRA curve was also highest for multicomponent interventions (1.00), followed by MBIs (0.754), CBT (0.622), and counseling (0.520), suggesting that multicomponent interventions could be the optimal options for managing perinatal symptoms of stress (**Supplementary Figure 3**, **Supplementary Table 3**). Again, it is worth noting that only one study on multicomponent interventions and two studies on counseling were included in the analysis; as a result, the reported effect sizes were subject to a low level of generalizability.

Between-group comparisons across the six interventions showed that multicomponent interventions could produce better effects compared to all the other interventions. In addition, MBIs and CBT were found to be more effective compared to psychoeducation (SMD=-0.99, 95% CI: -1.81 to -0.17 and SMD=-0.75, 95% CI: -1.51 to -0.01, respectively) (**Table 4**).

**Table 4:** Relative effects of the different prenatal psychotherapies and psychosocial interventions for stress (Random model).

Multicomponent interventions						
-4.55(-6.38, -2.72)*	MBI					
-4.79 (-6.61, -2.97)*	-0.24 (-0.88, 0.40)	CBT				
-5.54 (-7.43, -3.66)*	-0.99 (-1.81, -0.17)*	-0.75 (-1.51, -0.01)*	Psychoeducation			
-4.99 (-7.02, -2.96)*	-0.44 (-1.55, 0.67)	-0.20 (-1.29, 0.89)	0.55 (-0.65, 1.76)	Counseling		
-5.66 (-7.90, -3.42)*	-1.11 (-2.56, 0.34)	-0.87 (-2.31, 0.57)	-0.12 (-1.64, 1.41)	-0.67 (-2.37, 1.03)	IPT	
-5.82 (-7.58, -4.05)*	-1.26 (-1.74, -0.79)*	-1.03 (-1.45, -0.60)*	-0.27 (-0.94, 0.39)	-0.83 (-1.83, 0.18)	-0.16 (-1.53, 1.21)	Control

*P<0.05; MBI, Mindfulness-based intervention; CBT, Cognitive behavioral therapy; IPT=Interpersonal treatment.

### 3.5 Meta-regression and sensitivity analyses

The results of the meta-regression analyses suggested a significant association between the geographic region of origin of the studies and the effects of prenatal CBT on depressive symptoms, anxious symptoms and stress (**Supplementary Table 4**). Specifically, prenatal CBT studies originating from Asian countries/regions tended to produce greater effect sizes than those originating from other regions. Further subgroup analyses showed that prenatal CBT could reduce symptoms from depressive symptoms, anxious symptoms and stress by an SMD of -1.04 (-1.65 to -0.43), -1.14 (-1.45 to -0.83) and -1.42 (-1.81 to -1.04) among Asian pregnant women, while these figures among non-Asian pregnant women were -0.23 (-0.46 to -0.01), -0.28 (-0.46 to -0.09), and -0.38 (-0.69 to -0.07), respectively.

Sensitivity analysis was conducted by removing the studies with a high risk of bias so as to explore their potential influence on the synthesized effects, and the results showed good agreement before and after removing these studies individually, indicating robust findings.

### 3.6 Certainty of evidence assessment

The certainty of evidence for each network estimate was determined using the CINeMA approach. As shown in **Supplementary Table 5**, the certainty of evidence for the majority of the comparisons was rated low due to concerns about within-study bias and heterogeneity.

### 3.7 Publication bias

The funnel plots exhibited substantial symmetry across all outcome indicators (**Supplementary Figure 4**), and the corresponding Egger’s test values were nonsignificant (*P* > 0.05), suggesting that the synthesized results were less likely to be influenced by publication bias.

## 4 Discussion

Multiple psychotherapies and psychosocial interventions have been recommended for the management of perinatal psychological disorders by international and national guidelines. However, the synthesized effect size of these strategies and their relative effectiveness remain unclear. In the current study, this research gap was addressed by performing network meta-analyses. The results showed that prenatal psychotherapies and psychosocial interventions tend to be effective in reducing symptoms of depressive symptoms, anxious symptoms and stress, with SMDs of -0.70, -0.81, and -1.05, respectively. Moreover, the results suggest that multicomponent interventions, counseling, and MBIs may be superior options for the management of depressive symptoms, anxious symptoms and stress, respectively.

### 4.1 Prenatal psychotherapies and psychosocial interventions are effective in reducing symptoms of depression

In addition to demonstrating beneficial synthesized efficacy, the results of this study show that all five of the examined interventions are effective in reducing symptoms of depression, including multicomponent interventions, IPT, CBT, MBIs, and psychoeducation. In the included trials, CBT was the most frequently utilized psychosocial intervention for perinatal symptoms of depression and demonstrated a moderate effect size (SMD=-0.69). This finding aligns with the recommendations of several international and national guidelines that endorse CBT as a first-line treatment for perinatal depression due to its evidence-based efficacy and adaptability to the unique needs of this population (26, 27). MBIs are another commonly utilized strategy in the management of perinatal depression, demonstrating moderate efficacy (SMD=-0.68). To date, there exists a substantial body of evidence supporting the effectiveness of MBIs in alleviating perinatal depressive symptoms (28–31). Of note, a recent meta-analysis encompassing 25 RCTs suggested that MBIs may be a valuable adjunct to existing intervention options (29). Moreover, we found that IPT is an effective intervention for alleviating symptoms of depression, with a moderate effect size of -0.75, which is consistent with the results of several previous traditional meta-analyses (31–33). Specifically, a systematic review of 28 studies reported that IPT was effective in reducing symptoms of depression in both preventive and treatment studies, providing further support for the robustness of this intervention in the context of perinatal depression management (32). Although a statistically significant effect of psychoeducation was demonstrated, its effect size was quite small (SMD=-0.14). This finding aligns with previous research. For instance, a mixed-methods systematic literature review encompassing 20 studies reported a small reduction in symptoms of depression (SMD = 0.32) when psychoeducation was used as the core intervention (34). This highlights the potential variability in the effectiveness of psychoeducation based on its implementation context, underscoring the need for further research to clarify its effective modality.

Remarkably, both the synthesized effect size and the P-analysis results indicated that multicomponent interventions, which incorporated psychoeducation, PST, MBI techniques, IPT, CBT, biofeedback therapy, situation simulation training, peer support, and integrated social support, could be the most effective approach for alleviating depressive symptom scores. While no conventional meta-analyses have specifically examined the efficacy of multicomponent interventions for perinatal depression, existing empirical evidence is consistent with the current review in supporting their potential (35–39). In addition, a systematic review demonstrated the effectiveness of these interventions in addressing complex psychological symptoms, such as substance abuse. Waqas et al. also highlighted the need for targeted, multi-component strategies to address the heterogeneity of perinatal depressive symptoms (40, 41).

### 4.2 Prenatal psychotherapies and psychosocial interventions are effective in reducing symptoms of anxiety

In addition to a synthesized efficacy, the results of meta-analyses demonstrated the favorable effects of ACT, IPT, counseling, multicomponent interventions, and CBT in relieving symptoms of anxiety. Counseling interventions were found to have a large effect size in reducing symptoms of anxiety (SMD=-1.13), which is consistent with an existing systematic review (42). The U.S. Preventive Services Task Force recommends that clinicians refer individuals at risk for perinatal mood disorders to counseling interventions, emphasizing their efficacy and minimal risk to patients (42). Large effect sizes were also demonstrated for multicomponent interventions (SMD=-0.86) and CBT (SMD=-0.81), which agrees with a systematic review and meta-analysis of 79 RCTs. The review found that CBT could reduce perinatal anxiety by an SMD of -0.63 and -0.62, when used independently or as part of a multicomponent intervention (43). These findings underscore the robust efficacy of CBT and multicomponent approaches in addressing perinatal mental health concerns.

Despite their relatively larger effect sizes, the efficacy of ACT and IPT is not guaranteed, because only one study on ACT and one study on IPT were included in our analysis. Nonetheless, ACT has been suggested as a potentially beneficial treatment for moderate-to-severe perinatal mental health problems owing to its trans-diagnostic nature (44). Consequently, further high-quality RCTs are warranted to explore the effectiveness of ACT in addressing perinatal mental health disorders.

### 4.3 Prenatal psychotherapies and psychosocial interventions are effective in reducing stress

Although the pooled efficacy is significant, different types of psychotherapies and psychosocial interventions showed distinct effectiveness: multicomponent interventions, MBIs, CBT, and counseling tended to be effective strategies for the management of perinatal stress, while psychoeducation and IPT were ineffective. Meta-analyses suggest that MBI could produce a very large effect size in lowering perinatal stress, with an SMD of -1.31. This result is inconsistent with the findings of an existing systematic review. The review concluded that MBI significantly promoted stress remission in perinatal women with pre-existing mental health conditions, but had no effect on those without such conditions (30). This discrepancy may be attributed to the heightened psychological needs of women with mental health issues, who may benefit more from mindfulness practices due to their potential to alleviate distress and enhance resilience. This underscores the importance of tailoring interventions to specific populations based on their psychological profiles. CBT was found to have a large effect size (SMD=-1.03) in relieving perinatal stress. The finding is consistent with a previous systematic review and meta-analysis, which confirmed the efficacy of CBT in reducing perinatal stress, reporting a significantly large effect size (SMD=-0.96) (43). These results collectively identify CBT as a robust intervention for addressing perinatal stress, reinforcing its value as an intervention in this context.

However, while several pairwise meta-analyses demonstrated that psychoeducation was significantly more effective than the control interventions in reducing perinatal adverse psychological outcomes, this effect was not observed in the current network meta-analysis (34, 45). This discrepancy may be attributed to several factors, including small-study effects in pairwise analyses, heterogeneity introduced by indirect comparisons in network meta-analysis, and the adjustment for multiple comparisons in network meta-analyses, which can reduce the apparent significance of individual types of interventions, such as psychoeducation (25, 45, 46). Therefore, researchers are advised to prioritize outcomes derived from network meta-analyses, as they integrate direct and indirect evidence to provide a more comprehensive assessment of treatment effects while accounting for multiple comparisons.

Similarly, despite their encouraging effect size, the efficacy of multicomponent interventions and counseling is not guaranteed, as only one study on multicomponent interventions and two studies on counseling were included in the analysis. Further high-quality empirical evidence is indispensable before a sound conclusion can be drawn.

### 4.4 Meta-regression analyses

The results of meta-regression analyses show that the geographic region of origin of the studies is significantly associated with the effects of prenatal CBT on depressive symptoms, anxious symptoms and stress. This finding can be interpreted as the result of cultural differences. CBT, as a psychological intervention, is likely to be influenced by cultural context. For instance, cultural norms in the Asian population, including attitudes toward emotional expression, the stigma associated with mental health, and the preference for certain intervention approaches, may have enhanced the effectiveness of CBT (47). Therefore, these results should be interpreted with caution. They highlighted the value of investigating the potential role of cultural factors in prenatal psychotherapies and psychosocial interventions.

### 4.5 Strengths

To the best of our knowledge, this systematic review with network meta-analysis is among the very few pieces of evidence examining the synthesized efficacy of various prenatal psychotherapies and psychosocial interventions in the management of depressive symptoms, anxious symptoms and stress. This review also compared the relative effectiveness of different types of interventions. In strict adherence to the PRISMA guidelines, the methodology encompassed both qualitative and quantitative syntheses and performed sensitivity and meta-regression analyses to explore potential sources of heterogeneity and their influence on synthesized effectiveness. By systematically comparing the relative effectiveness of each intervention across these distinct yet interrelated conditions, this study not only added new insights to the current understanding of perinatal mental health management but also provided robust, evidence-based guidance for clinical decision-making.

### 4.6 Limitations

The interpretation of the findings needs to be considered in the context of several limitations. First, only studies published in English or Chinese were included in our analyses, which may have introduced publication bias and downgraded the generalizability of the findings. Second, the majority of the interventions were evaluated against non-active controls, limiting the availability of direct comparative evidence between different prenatal psychotherapies and psychosocial interventions. Although indirect comparisons between interventions were performed in this review, the lack of head-to-head comparisons may limit the robustness of the conclusions about their relative effectiveness. Third, as fourth-week follow-up data were not always available, the closest available time points were alternatively utilized for analysis in this situation. While this approach allowed for a more comprehensive synthesis of the available evidence, the results may have been influenced by inconsistent time points for outcome assessments. Fourth, the included studies were subject to substantial risk of bias, and the certainty of evidence for the majority of comparisons was rated as low, which would raise concerns about the soundness of the conclusions. Finally, although the effectiveness of prenatal multicomponent interventions appears to be promising, especially for decreasing symptoms of depression and anxiety. However, the optimal modality and duration of intervention remain undefined due to the high heterogeneity among the included studies. Healthcare providers need to make individualized clinical decisions according to clients’ preferences and regular feedback.

### 4.7 Implications for clinical practice and further studies.

The results of this study confirmed that prenatal psychotherapies and psychosocial interventions tend to be effective in alleviating depressive symptoms, anxious symptoms and stress, with moderate to large effect sizes. Specifically, multicomponent interventions, counseling, and MBIs could be superior options for the management of depressive symptoms, anxious symptoms and stress, respectively. Additionally, CBT tends to be a reasonable option that can produce a satisfactory, beneficial effect on all of the aforementioned prenatal psychological issues. However, the effects of psychoeducation appear to be limited, and therefore, it is suggested that psychoeducation be incorporated into multicomponent interventions rather than utilized independently in clinical practice.

Considering the potential influence of the high risk of bias in the included studies on the synthesized evidence, further trials with robust research designs and strict quality control are valuable. While our findings suggest potential benefits under certain conditions, future studies should explore the underlying mechanisms of these interventions and identify factors that influence treatment response and efficacy, and client acceptability. These investigations will contribute to optimizing the effectiveness and cost-effectiveness of these approaches. Additionally, given the insufficient evidence on the sustainability of intervention effects, future research should include longer follow-up periods to evaluate the long-term effectiveness. Furthermore, future trials should systematically explore participants’ views, experiences, and motivations for adhering to or discontinuing interventions. These insights will be instrumental in enhancing intervention efficacy and minimizing attrition rates. Finally, to enhance the accessibility and acceptance of perinatal psychological interventions, future studies are encouraged to explore more user-friendly intervention delivery formats, such as digital platforms or artificial intelligence-assisted approaches.

## 5 Conclusion

The results of this systematic review with network meta-analysis suggest that prenatal psychotherapies and psychosocial interventions tend to be effective in reducing depressive symptoms, anxious symptoms and stress. More specifically, multicomponent interventions, counseling, and MBIs may be superior options for the management of depressive symptoms, anxious symptoms and stress, respectively, while CBT tends to be a reasonable option that can produce a satisfactory beneficial effect on all of these prenatal psychological problems. Further studies with robust research designs and strict quality control conducted in various populations are desirable to draw firmer conclusions.
