# Supplementary material for: Effects of prenatal psychotherapies and psychosocial interventions on depressive symptoms, anxious symptoms and stress: a systematic review and network meta-analysis
Source: Front Psychiatry. 2026 Jan 28;16:1624924. doi: 10.3389/fpsyt.2025.1624924 (PMC12890675; doi:10.3389/fpsyt.2025.1624924)
Supplement: Supplementary file 1 [file DataSheet1.zip › 新建文件夹/Supplementary Figure 4. Funnel plots for the meta-analysis.pdf]

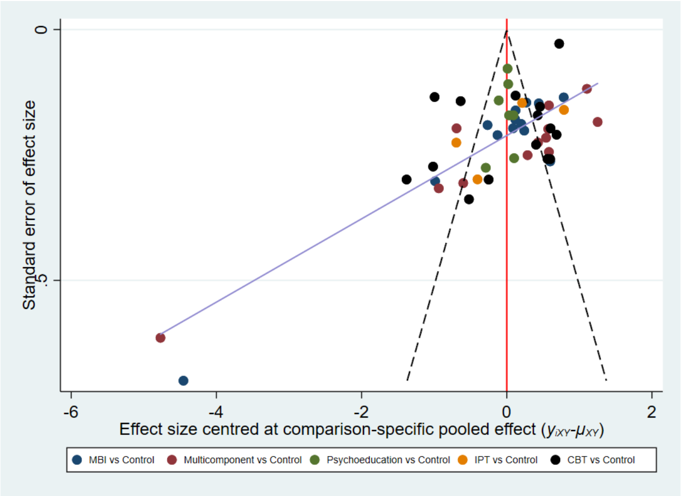

(1) Funnel plots for the meta-analysis of depressive symptoms

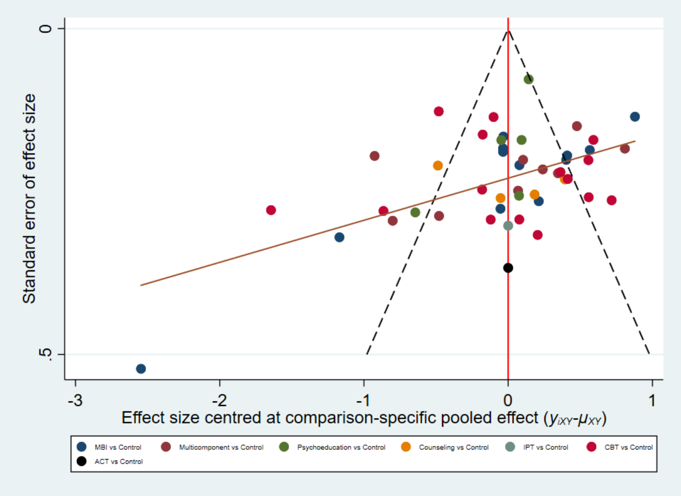

(2) Funnel plots for the meta-analysis of anxious symptoms

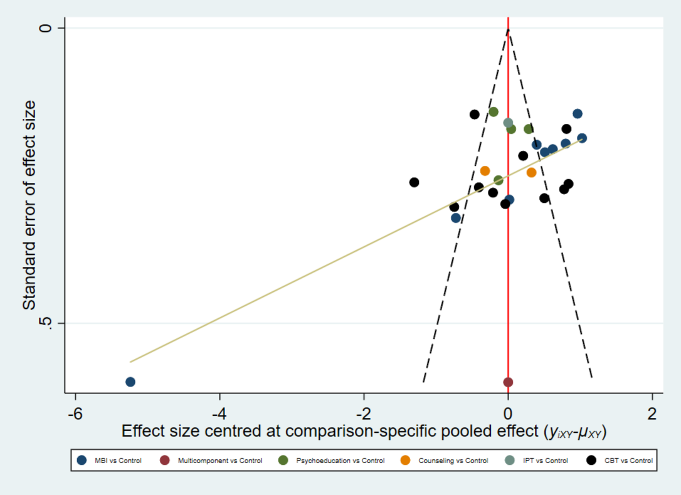

(3) Funnel plots for the meta-analysis of stress
