# Supplementary material for: Effects of prenatal psychotherapies and psychosocial interventions on depressive symptoms, anxious symptoms and stress: a systematic review and network meta-analysis
Source: Front Psychiatry. 2026 Jan 28;16:1624924. doi: 10.3389/fpsyt.2025.1624924 (PMC12890675; doi:10.3389/fpsyt.2025.1624924)
Supplement: Supplementary file 1 [file DataSheet1.zip › 新建文件夹/Supplementary Table 1. The detailed search strategies for different databases.docx]

Supplementary Table 1. The detailed search strategies for different databases

| **PubMed** | |
| --- | --- |
| #1 | (((((Care, Prenatal OR Antenatal Care OR Care, Antenatal OR Prenatal Care) OR ("Pregnancy"[Mesh]OR Pregnancies OR Gestation)) OR ((((((((antepartum period[Title/Abstract]) OR (prenatal*[Title/Abstract])) OR (pre-natal[Title/Abstract])) OR (antenatal women[Title/Abstract])) OR (antenatal*[Title/Abstract])) OR (antepartum[Title/Abstract])) OR (pregnant[Title/Abstract])) OR (pregnant*[Title/Abstract]))) AND (Clinical Trial, Randomized Controlled Trial) |
| #2 | (((((("Mental Health"[Mesh] OR Health, Mental OR Mental Hygiene OR Hygiene, Mental) OR ("Psychology"[Mesh] OR Side Effects, Psychological OR Psychological Side Effect OR Side Effect, Psychological OR Psychological Side Effects OR Psychosocial Factors OR Factor, Psychosocial OR Factors, Psychosocial OR Psychosocial Factor OR Psychological Factors OR Factor, Psychological OR Psychological Factor OR Factors, Psychological OR Psychologists OR Psychologist)) OR ("Depression"[Mesh] OR Depressive Symptoms OR Depressive Symptom OR Symptom, Depressive OR Symptoms, Depressive OR Emotional Depression OR Depression, Emotional)) OR ("Psychophysiology"[Mesh] OR Psychology, Physiologic OR Physiological Psychology OR Psychology, Physiological OR Physiologic Psychology OR Physiologic Psychologies OR Psychologies, Physiologic OR Mind-Body Relations (Physiology) OR Mind Body Relations (Physiology) OR Mind-Body Relation (Physiology) OR Relation, Mind-Body (Physiology) OR Relations, Mind-Body (Physiology) OR Mind-Body Relationship (Physiology) OR Mind Body Relationship (Physiology) OR Mind-Body Relationships (Physiology) OR Relationship, Mind-Body (Physiology) OR Relationships, Mind-Body (Physiology))) OR ("Psychotherapy"[Mesh] OR Psychotherapies)) OR (((((((((((((((((depress*[Title/Abstract]) OR (well-being[Title/Abstract])) OR (wellbeing[Title/Abstract])) OR (well being[Title/Abstract])) OR (stress[Title/Abstract])) OR OR (mental health[Title/Abstract])) OR (emotion*[Title/Abstract])) OR (psycholog*[Title/Abstract])) OR (panic[Title/Abstract])) OR (anxiety[Title/Abstract])) OR (anxious[Title/Abstract])) OR (mood[Title/Abstract])) OR (fear of birth[Title/Abstract])) OR (fear of child birth[Title/Abstract])))) AND (Clinical Trial, Randomized Controlled Trial) |
| #3 | (((((psychosocial intervention[Mesh])) OR (psychosocial[Title/Abstract])) OR OR behaviour therapy[Title/Abstract])) OR (cognitive therapy[Title/Abstract])) OR (cognition[Title/Abstract])) OR (cognitive psychotherapy[Title/Abstract])) OR (cognition therapy[Title/Abstract])) OR (behavioral therapy[Title/Abstract])) OR (cognitive arousal[Title/Abstract])) OR (cognitive behavioral[Title/Abstract])) OR (cognitive behavioural[Title/Abstract])) OR (CBT[Title/Abstract])) OR (ICBT[Title/Abstract])) OR (cognitive restructuring[Title/Abstract])) OR (psychoeducation[Title/Abstract])) OR (behaviour modification[Title/Abstract])) OR (cognitive remediation[Title/Abstract])) OR (interpersonal therapy[Title/Abstract])) OR (acceptance commitment therapy[Title/Abstract])) OR (mindfulness[Title/Abstract])) OR (problem-solving[Title/Abstract])))) |
| #4 | #1 AND #2 AND #3 |
| **EMBASE** | |
| #1 | ‘Prenatal Care’/exp OR ‘Pregnancy*’:ti,ab OR ‘Antepartum period*’:ti,ab OR prenatal*:ti,ab OR pre-natal*:ti,ab OR antenatal*:ti,ab OR ante-natal*:ti,ab OR antepartum:ti,ab OR ante-partum*:ti,ab OR pregnant:ti,ab OR pregnant*:ti,ab |
| #2 | ‘mental health’/exp OR ‘psychology’/exp OR ‘depression’/exp OR ‘psychophysiology’/exp OR ‘psychotherapy’/exp OR ‘depress*’:ti,ab OR ‘well*being’:ti,ab OR ‘stress’:ti,ab OR ‘psychosocial’:ti,ab OR ‘mental health’:ti,ab OR ‘emotion*’:ti,ab OR ‘psycholog*’:ti,ab OR ‘anxiety’:ti,ab OR ‘anxious’:ti,ab OR ‘mood’:ti,ab OR ‘fear of birth’:ti,ab OR ‘fear of child birth’:ti,ab |
| #3 | ‘behaviour therapy’:ti,ab OR ‘cognitive therapy’:ti,ab OR psychotherapy:ti,ab OR ‘behavioural activation’:ti,ab OR ‘cognitive behaviour’:ti,ab OR cognition:ti,ab OR ‘cognitive psychotherapy’:ti,ab OR ‘cognitive behavioural’:ti,ab OR CBT:ti,ab or ICBT:ti,ab OR ‘cognitive restructuring’:ti,ab OR psychoeducation:ti,ab OR ‘behaviour modification’:ti,ab OR ‘cognitive remediation’:ti,ab OR ‘interpersonal therapy’:ti,ab OR ‘acceptance commitment therapy’:ti,ab OR ‘mindfulness’:ti,ab OR ‘problem-solving’:ti,ab |
| #4 | #1 AND #2 AND #3 |
| **Cochrane Central Register of Controlled Trials** | |
| #1 | MeSH descriptor: [Prenatal Care] explode all trees OR MeSH descriptor: [Pregnancy] explode all trees OR (prenatal* OR pre-natal* OR antenatal* OR ante-natal* OR antepartum OR ante-partum* OR pregnant OR pregnan*):ti,ab,kw |
| #2 | MeSH descriptor: [Mental health] explode all trees OR MeSH descriptor: [Psychology] explode all trees OR MeSH descriptor: [Depression] explode all trees OR MeSH descriptor: [Psychophysiology] explode all trees OR MeSH descriptor: [psychotherapy] explode all trees OR MeSH descriptor: [anxiety] explode all trees OR MeSH descriptor: [anxiety disorder] explode all trees OR (Depress* OR well-being OR stress OR psychosocial OR mental health OR emotion* OR psycholog* OR panic OR anxiety OR anxious OR mood OR fear of birth OR fear of child birth):ti,ab,kw |
| #3 | MeSH descriptor: [Cognitive behavioral therapy] explode all trees OR MeSH descriptor: [Mindfulness] explode all trees OR (behaviour therapy OR cognitive therapy OR Psychotherapy OR behavioural activation OR cognitive behaviour OR cognition OR cognitive psychotherapy OR cognition therapy OR behavioral therapy OR cognitive arousal OR cognitive behavioral OR cognitive behavioural OR CBT OR ICBT OR cognitive restructuring OR psychoeducation OR behaviour modification OR cognitive remediation OR interpersonal therapy OR acceptance commitment therapy OR mindfulness OR problem-solving):ti,ab,kw |
| #4 | #1 AND #2 AND #3 |
| **PsycINFO** | |
| #1 | TX prenatal care OR TX pregnancy OR TX Antepartum OR TX Prenatal OR TX pre-natal OR TX perinatal OR TX antenatal OR TX ante-natal OR TX ante-partum OR TX pregnant |
| #2 | TX Mental Health OR TX Psychology OR TX Depression OR TX depress$ OR TX stress OR TX psychosocial OR TX emotion$ OR TX psycholog$ OR TX fear OR TX anxiety OR TX anxious OR TX fear of birth |
| #3 | TX ( cogniti$ or behaviour$ or psycho$ ) OR TX ( therap$ or activat$ or behavi$ or arous$ or restructur$ or modif$ or remediat$ ) OR TX ( CBT or ICBT ) OR TX interpersonal therapy OR TX risis oriented therapy OR TX acceptance commitment therapy OR TX mindfulness OR TX problem solving |
| #4 | TX ( rct or randomized control trial or randomized controlled trial or controlled trial ) |
| #5 | #1 AND #2 AND #3 AND #4 |
| **CINAHL** | |
| #1 | (MH “Antenatal Period+” OR “Antenatal Care+” OR “Perinatal Care”) OR TI(prenatal* OR pre-natal* OR antenatal* OR ante-natal* OR antepartum OR ante-partum* OR pregnan*) OR AB(prenatal* OR pre-natal* OR OR antenatal* OR ante-natal* OR antepartum OR ante-partum* OR pregnan*) |
| #2 | (MH “Mental Health” OR “Psychology+” OR “Depression+”) OR TI (Depress* OR stress OR psychosocial OR “mental health” OR emotion* OR psycholog* OR panic OR anxiety OR anxious OR mood OR fear OR “fear of birth” OR “fear of child birth”) OR AB (Depress* OR stress OR psychosocial OR “mental health” OR emotion* OR psycholog* OR panic OR anxiety OR anxious OR mood OR fear OR OR “fear of birth” OR “fear of child birth” ) |
| #3 | (MH “Cognitive Behavioral Therapy”) OR TI((((cogniti* OR behaviour* OR psycho*) N1 (therap* OR activat* OR behavi* OR arous* OR restructur* OR modif* OR remediat*)) OR CBT OR ICBT OR interpersonal therapy OR risis oriented therapy OR acceptance commitment therapy OR mindfulness OR problem-solving )) OR AB((((cogniti* OR behaviour* OR psycho*) OR (therap* OR activat* OR behavi* OR arous*)) OR CBT OR ICBT) OR interpersonal therapy OR acceptance commitment therapy OR mindfulness OR problem-solving) |
| #4 | #1 AND #2 AND #3 |
| **Scoups** | |
| #1 | TITLE-ABS-KEY ("prenatal care" OR pregnancy OR "antepartum period" OR prenatal* OR pre-natal* OR antenatal* OR antepartum OR ante-partum OR pregnant OR pregnan*) |
| #2 | TITLE-ABS-KEY (depress* OR "well?being" OR stress OR psychosocial OR "mental health" OR emotion* OR psycholog* OR fear OR anxiety OR mood OR "fear of birth" OR "fear of child birth") |
| #3 | "behavio?r therapy" OR "cognitive therapy" OR psychotherapy OR "behavio?ral activation" OR "cognitive behavio?r therapy" OR "cognitive arousal" OR "cognitive behavio?ral" OR cbt OR icbt OR "cognitive restructuring" OR psychoeducation OR "behavio?r modification" OR "cognitive remediation" OR psychoeducation OR "behavio?r modification" OR "cognitive remediation" OR (CBT OR ICBT) OR interpersonal therapy OR acceptance commitment therapy OR mindfulness OR problem-solving |
| #4 | #1 AND #2 AND #3 |
| **Web of science** | |
| #1 | TS=("prenatal care" OR "pregnancy" OR "antepartum period" OR "pre?natal*" OR "ante?natal*" OR antepartum OR "ante-partum*"OR pregnant OR pregnan*) |
| #2 | TS=("mental health" OR "psychology" OR "Depress*" OR "psychophysiology" OR "psychotherapy" OR "well?being" OR stress OR psychosocial OR emotion* OR psycholog* OR fear OR anxiety OR anxious OR mood OR "fear of birth" OR "fear of child birth") |
| #3 | TS=(“behavio?r therapy” OR “cognitive therapy” OR psychotherapy OR “behavior?r activation” OR “cognitive behavior?r” OR cognition OR “cognitive psychotherapy” OR “cognitive therapy” OR “behavior?r therapy” OR “cognitive arousal” OR “cognitive behavior?ral” OR CBT OR ICBT OR “cognitive restructuring” OR psychoeducation OR “behavior?r modification" OR “cognitive remediation” OR interpersonal therapy OR acceptance commitment therapy OR mindfulness OR problem-solving) |
| #4 | TS=("intervention study" OR "Randomi?zed controlled trails as topic" OR "randomi?zed controlled trail" OR clinical trails, randomi?ed OR trials, randomi?ed clinical OR controlled clinical trail, randomi?ed OR "clinical trials as topic" OR "Clinical trail") |
| #5 | #1 AND #2 AND #3 AND #4 |
| **ProQuest Dissertation and Theses** | |
| #1 | Summary("prenatal care" OR pregnancy OR "antepartum period" OR "pre?natal$" OR antenatal$ OR antepartum OR ante-partum$ OR pregnant OR “pregnan$”) |
| #2 | Summary("mental health" OR psychology OR depress* OR psychophysiology OR psychotherapy OR "well?being" OR stress OR psychosocial OR emotion* OR psycholog* OR anxiety OR anxious OR mood OR fear OR "fear of birth" OR "fear of child birth") |
| #3 | Summary(“behavior?r therapy” OR “cognitive therapy” OR Psychotherapy OR “behavior?ral activation” OR cognitive behavior?r OR cognition OR “cognitive psychotherapy” OR “cognition therapy” OR “behavior?ral therapy” OR “cognitive arousal” OR “cognitive behavior?ral” OR CBT OR ICBT OR “cognitive restructuring” OR psychoeducation OR “behavio?r modification” OR “cognitive remediation” OR interpersonal therapy OR acceptance commitment therapy OR mindfulness OR problem-solving) |
| #4 | Summary("intervention study" OR "Randomi?zed controlled trails as topic" OR "randomi?zed controlled trail" OR clinical trails, randomi?ed OR trials, randomi?ed clinical OR controlled clinical trail, randomi?ed OR "clinical trials as topic" OR "Clinical trail") |
| #5 | #1 AND #2 AND #3 AND #4 |
| **China National Knowledge Infrastructure(CNKI)** | |
| #1 | TI= (‘怀孕’ OR ‘孕妇’ OR ‘妊娠’ OR ‘孕期’ OR ‘产前’ OR ‘分娩前’) |
| #2 | TI=(‘心理’OR ‘精神’ OR ‘健康’OR ‘抑郁’OR ‘情绪’OR ‘压力’OR ‘焦虑’+ ‘恐惧’) |
| #3 | TI=(‘认知’OR ‘认知行为’OR ‘认知行为疗法’OR ‘行为’OR ‘社会心理’OR ‘正念’OR ‘问题解决’OR ‘接纳承诺’) |
| #4 | TKA=（ ‘试验’OR ‘RCT’ OR ‘随机对照’OR ‘干预’OR ‘对照’OR ‘效果’） |
| #5 | #1 AND #2 AND #3 AND #4 |
| **Wan Fang Database for Chinese Periodicals** | |
| #1 | TI（（怀孕） OR （孕妇） OR （妊娠） OR （孕期） OR （产前） OR （分娩前）） |
| #2 | TI（（心理）OR （精神） OR （心理 ）OR （健康） OR （抑郁） OR （情绪） OR （压力） OR （焦虑） OR （恐惧） ） |
| #3 | TI, KW（（认知行为） OR （认知行为疗法）OR （认知） OR （行为）OR （社会心理） OR （正念） OR （问题解决） OR （接纳承诺）） |
| #4 | AB (（试验） OR （RCT ）OR （随机对照） OR （干预） OR （对照 ）OR （效果）) |
| #5 | #1 AND #2 AND #3 AND #4 |
| **VIP Database for Chinese Technical periodicals** | |
| #1 | TI= （怀孕 OR 孕妇OR 妊娠 OR 孕期 OR 产前 OR 分娩前) |
| #2 | TI=（心理 OR 精神OR 心理 OR 健康 OR 抑郁OR 情绪OR 压力OR 焦虑OR 恐惧） |
| #3 | TI, KW=(认知 OR 认知行为 OR 认知行为疗法 OR 行为OR 社会心理 OR 正念 OR 问题解决 OR 接纳承诺) |
| #4 | AB= (试验 OR RCT OR 随机对照 OR 干预 OR 对照 OR 效果) |
| #5 | #1 AND #2 AND #3 AND #4 |
