# Supplementary material for: Effects of prenatal psychotherapies and psychosocial interventions on depressive symptoms, anxious symptoms and stress: a systematic review and network meta-analysis
Source: Front Psychiatry. 2026 Jan 28;16:1624924. doi: 10.3389/fpsyt.2025.1624924 (PMC12890675; doi:10.3389/fpsyt.2025.1624924)
Supplement: Supplementary file 1 [file DataSheet1.zip › 新建文件夹/Supplementary Table 2. Details of psychotherapies and psychosocial interventions of the included studies.docx]

Supplementary Table 2. Details of psychotherapies and psychosocial interventions of the included studies

| Primary author, publication year  Country/Region | Aim | Intervener | Outline | Platform | Regime | | | | |
| --- | --- | --- | --- | --- | --- | --- | --- | --- | --- |
|  |  |  |  |  | No. of sessions | Session duration | frequency | Intervention period | Stage of assessment |
| Abdollahi, 2020  Iran | Prevention | Therapist | (1) Stages of change; (2) maternal feelings and ambivalences; (3) Positive and negative aspects of behavior change; (4) values and goals; (5) dangerous situation and tempting return | Face-to-face, group-based | 5 | 120 mins | Weekly | 5 weeks | T0: Baseline  T1: Post-intervention |
| Abujilban, 2024  Jordan | Treatment | Therapist | Orient and explain the therapy process and structure; collect information and assess the problem; review | Telephone | 7 | 30 mins | Twice a week | 4 weeks | T0: Baseline  T1: Post-intervention |
| Alipour, 2020  Iran | Prevention | Therapist | Focused on the re-establishment of appropriate communication between partners, including the establishment of a good relationship and creation of an atmosphere of trust and security, understanding the changes and psychological needs of pregnant women, the importance of communication skills, psychological health during pregnancy, the description of the differences between men and women, common problems in the family, training of self-awareness and self-care skills, and the assessment of the communication barriers in them | Face-to-face, group-based | 7 | 120 mins | Twice a week | 4 weeks | T0: Baseline  T1: Post-intervention  T2: 3-month follow-up |
| Baniaghil, 2022  Iran | Prevention | Therapist | The content included teaching principles and exercises that develop being in the present moment with acceptance and teach individuals to have a nonjudgmental attitude. These pieces of training lead to relief from distracting thoughts and help create nonjudgmental alertness, alternative attitudes and behaviors, and appropriate responses to complex situations. | Face-to-face, group-based | 8 | 120-150 mins | Weekly | 8 weeks | T0: Baseline  T1: Post-intervention |
| Bayat, 2021  Iran | Prevention | Therapist | Expression of clients’ feelings about the result of screening and relaxation training; misconceptions about the cause of screening anxiety; A-B-C chain training; Helping women identify their intellectual errors and examine them. Mindfulness training, strengthening positive thoughts, and relaxation training | Face-to-face | 4 | 45-60 mins | Twice a week | 2 weeks | T0: Baseline  T1: Post-intervention |
| Bittner, 2014  Germany | Prevention | Therapist | It consists of psychoeducation, introduction to cognitive behavioral strategies, performance of exercises/role-playing, and progressive muscle relaxation. | Face-to-face, group-based | 8 | 90 mins | NR | NR | T0: Baseline  T1: Post-intervention  T2: 3-month postpartum |
| Chan, 2019  Hong Kong | Prevention | Therapist | Information about nutrition, infant care, and vaccine injections for infants, as well as videos demonstrating what expectant mothers might face when delivering their baby, were delivered off-site, organized by topic to increase usability, and open to access at anytime and anywhere for assigned users. | Online | NR | NR | NR | NR | T0: Baseline  T1: 4-week postpartum |
| Cui, 2023  China | Treatment | Therapist | Spousal co-participation is to jointly assess pre-intervention concerns and psychological feelings during the intervention process, take courses on psychological knowledge, education courses on pregnancy and childbirth, and training on couple communication, followed by assisting pregnant women with behavioral activation and relaxation. | Face-to-face, group-based | 6 | 30-60 mins | NR | NR | T0: Baseline  T1: Post-intervention |
| Esfandiari, 2020  Iran | Prevention | Therapist | The program drew on supportive techniques, exploration, and expression. Addressing five pregnancy-related worries, including health problems and costs, parental responsibility, physical symptoms, infantile health, parenting, labor pain, and childbirth phobia. | Face-to-face, group-based | 6 | 120 mins | Weekly | 6 weeks | T0: Baseline  T1: 4-week post-intervention |
| Eteraf, 2023  Iran | Prevention | Therapist | Familiarity with stress and ways to control it, improve self-esteem and improve performance; Continue ways to control stress and increase adaptive skills | Face-to-face, group-based | 3 | 60-90 mins | Weekly | NR | T0: Baseline  T1: Post-intervention |
| Felder, 2020  USA | Treatment | Virtual therapist | Sleep restriction, stimulus control, cognitive therapy, relaxation techniques, sleep hygiene and education | Online | 6 | 20 mins | Weekly | 6 weeks | T0: Baseline  T1: Post-intervention (4-week after intervention)  T2: Follow up (12-week after intervention) |
| Forsell, 2017  Sweden | Treatment | Self-help | Customized mindfulness practices and topics relevant to perinatal women’s experiences. | Online | 8 | NR | Weekly | 8 weeks | T0: Baseline  T1: Post-intervention |
| Gao, 2012  China | Prevention | Therapist | Using specific IPT techniques (information giving, clarification, communication analysis, role-play and brainstorming) to help women focus on the transition to motherhood and develop social support for resolving interpersonal conflict and issues | Face-to-face, group-based | 2 antenatal IPT sessions, and a telephone follow-up within 2 weeks after delivery | 90 mins | NR | NR | T0: Baseline  T1: Post-intervention (6-week postpartum)  T2: 3-month postpartum |
| Gedde-Dahl, 2012  Norway | Prevention | Self-help | The CD contained an introduction track and three relaxation tracks: one with relaxing music and guided imagery of the birth process, one with relaxing music and guided positive affirmations and one with relaxing music only. | Not applicable | NR | NR | NR | NR | T0: Baseline  T1: Post-intervention (During and post-delivery) |
| Golshani, 2021  Iran | Treatment | Therapist | Identifying problems, introducing CBT and cognitive-behavioral patterns; conducting mood assessment, introducing progressive muscle relaxation and practicing it; introducing imagination and practicing it, and allocating homework assignments; imagination practices; introducing cognitive distortions; identifying hot thought; taking recurrence prevention process, introducing self-management sessions and its scheduling and allocating homework assignments | Face-to-face, group-based | 6 face-to-face courses and two telephone courses | 60-90 mins | Weekly | NR | T0: Baseline  T1: 4-week after intervention |
| Grote, 2009  USA | Treatment | Therapist | The intervention is based on principles of motivational interviewing and ethnographic interviewing and is designed to promote engagement by building trust and addressing the practical, psychological, and cultural barriers to care experienced by individuals who are socioeconomically disadvantaged. More specifically, during engagement, the interviewer elicits each participant’s unique barriers to care and engages in collaborative problem solving to ameliorate each barrier | Face-to-face | 8 | NR | NR | NR | T0: Baseline  T1: 3-month postbaseline  T2: 6-month postpartum |
| Hassdenteufel, 2023  Germany | Treatment | Therapist | The intervention involving psychoeducational and obstetrical  content, mindfulness exercises, and cognitive behavioral approaches.  The psychoeducational content encompassed the occurrence of pregnancy-related stress, emergence of mental vicious circles, and individual sources of strength. Mediated skills comprised how to exit from the vicious circle of fear and the use of mindful breathing and mindful body scans | Online | 8 | 45 mins | Weekly | 8 weeks | T0: Baseline  T1: 2-week after baseline  T2: Mid-study (4-week after baseline)  T3: 6-week after baseline  T4: Immediately post-intervention  T5:1-month post-intervention  T6: 3-month post-intervention |
| Heller, 2022  Netherlands | Treatment | Therapist | A structured approach to solve (potentially) problems: write down a clear definition of the problems, generate multiple solutions, select the best solution, work out a systematic plan, carry out the solution, evaluate the implementation effect | Online | 5 | NR | Weekly | 5 weeks | T0: Baseline  T1: Post-intervention (10 weeks after baseline)  T2: 36 weeks’ gestation  T3: 6 weeks postpartum |
| Huang, 2015  China | Prevention | Therapist | Avoidance and control of possible stressors alleviate pregnant women’s negative perceptions and emotions during labor and delivery; Interviews to understand pregnant women’s cognitively related problems and to guide the development of positive coping styles; targeted relaxation training. | Offline | NR | NR | NR | NR | T0: Baseline  T1: Post-intervention |
| Hulsbosch, 2023  Netherlands | Treatment | Self-help | Stress and mindfulness; dealing with obstacles; the body and the sense; thoughts; emotions; communication and awareness; taking good care of yourself and your baby; the beginning of a new way of life | Online | 8 | 60 mins | Weekly | 8 weeks | T0: Baseline  T1: Mid-study  T2: Post-intervention  T3: 8-week follow-up |
| Jesse, 2015  USA | Prevention | Therapist | (1) introduction of the agenda and announcements; (2) an ice breaker activity; (3) a review of group rules (discussed in the first session); (4) a review of material covered in the previous sessions; (5) a presentation of the topic, (6) exercises related to the topic, and (7) a review of homework assignments to be completed between-sessions | Face-to-face, group-based | 6 | 120 mins | Weekly | 6 weeks | T0: Baseline  T1: Post-intervention  T2: 1-month follow-up |
| (1) Kaboli, 2017  Iran  (2) Salehi, 2020  Iran | Prevention | Therapist | (1) cognitive-behavioral consultation for pregnancy stress and tension based on Michelle G. Craske’s treatment guidelines Introduction to stress and coping methods; (2) subjective methods for coping; (3) physical methods for coping; (4) identifying ineffective thoughts; (5) reviewing; (6) problem-solving training | Face-to-face, group-based | 6 | 90 mins | NR | 6 weeks | T0: Baseline  T1: Post-intervention  T2: 3-week Follow up |
| Kalmbach, 2020  USA | Prevention | Virtual therapist | Behavior components (sleep restriction, stimulus control), cognitive components (eg, cognitive restructuring, paradoxical intention), progressive muscle relaxation, and sleep hygiene | Online | 6 | NR | Weekly | 6 weeks | T0: Baseline  T1: Post-intervention  T2: 6-week postpartum |
| Khamseh, 2019  Iran | Treatment | Therapist | Based on IDEAL model (identify, define, explore, act, look back) | Face-to-face, group-based or individual | 5 | 90 mins | Weekly | 5 weeks | T0: Baseline  T1: Post-intervention  T2:4-week follow-up |
| Kharaghani, 2023  Iran | Prevention | Therapist | Using CBT cognitive correction technique to reduce anxiety and worry; Teaching problem-solving and self-efficacy skills to deal with avoided situations and keep value positive traits | Telephone | 4 | 90-120mins | Twice a week | 2 weeks | T0: Baseline  T1: Post-intervention  T2:6-week follow-up |
| Khatibi, 2021  Iran | Prevention | Therapist | (1) providing a brief introduction to CBT; (2) delivering the treatment rationale, sleep training, behavioral therapy, and bedtime guidelines and recommendation; (3)correcting modifying cognitive behavioral errors, sleep hygiene education and treatment; (4)adherence to treatment, expressing cognitive rationality, presenting teaching techniques of constructive anxiety; (5)adherence to treatment, removing any problem on cognitive and behavioral component, summary | Face-to-face, group-based | 5 | 90 mins | weekly | 5 weeks | T0: Baseline  T1: Post-intervention  T2: 8-week follow-up |
| Khorsandi, 2015  Iran | Prevention | Therapist | The educational intervention in this study was designed to educate the subjects about the interactive nature of stress and how to deal with it; methods of self-control; identification of thoughts, images, feelings, and behaviors; and non-adaptive undetected compromise evaluations in order to facilitate compatibility and compliance. | Face-to-face, group-based | 8 | 90 mins | Weekly | 8 weeks | T0: Baseline  T1: Post-intervention |
| Kozinszky, 2012  Hungary | Prevention | Therapist | Patient education; PPD screening and coping skills; recognizing distress and seeking help; recapitulation and relaxation | Face-to-face, group-based | 4 | 180 mins | Weekly | 4 weeks | T0: Baseline  T1: 6-week postpartum |
| Kuo, 2022  Taiwan | Treatment | Therapist | Topics covered in the childbirth sessions included physiological and psychological changes during pregnancy, labor process and care during the intrapartum period, relaxation and breathing, pain relief during labor, breastfeeding, newborn care, and postpartum care. Mindfulness sessions included the practice of mindfulness breathing, body scans, body stretching, sitting meditation, and mindfulness walking. | Face-to-face, group-based | 8 | 120 mins | Weekly | 8 weeks | T0: Baseline  T1: Post-intervention (36-week gestation)  T2: 1-week postpartum |
| Le, 2011  USA | Prevention | Therapist | Introduction; Activities and my mood; Activities and my baby’s mood; Pleasant activities help construct a healthy reality for my baby and me; Thoughts and my mood; How to give me good advice; Fighting harmful thoughts that affect my baby and me; Thoughts I want to learn to teach my baby; Contact with others and my mood; How to get support for me and my baby; The importance of good communication and preparing for my baby’s birth; Using the course to build a healthy life for me and my baby | Face-to-face, group-based | 8 | 120 mins | Weekly | 8 weeks | T0: Baseline  T1: Post-intervention  T2: 6-week postpartum  T3: 4-month postpartum  T4: 1-year  postpartum |
| Leung, 2012  Hong Kong | Prevention | Therapist | COPE included (1) a short video clip to stimulate discussion. (2) participant identification of errors and development of alternative strategies; (3) role-play; and (4) weekly homework for practice. Each of the four sessions had a distinct, simple focus to ensure understanding and a clear take-home message. | Face-to-face, group-based | 4 | 90 mins | Weekly | 4 weeks | T0: Baseline  T1: Post-intervention  T2: 6-8week postpartum |
| Li, 2014  China | Treatment | Therapist | Introducing the rationale and principles of CBT, elucidating the relationship between emotion, behavior and cognition; introducing negative cognition, reconstructing correct cognitive patterns to help the pregnant women understand automatic thought patterns; homework assignments. | Face-to-face, group-based | 8 | 50 mins | Weekly | 8 weeks | T0: Baseline  T1: Post-intervention |
| Li, 2020  China | Prevention | Therapist | (1) The physiological and psychological changes in pregnancy, (2) coping methods for pregnancy stress, (3) relaxation techniques and, (4) family support | Face-to-face, group-based | 7 | 40-60 mins | Once every four weeks- biweekly | NR | T0: Baseline  T1: Post-intervention |
| Li, 2021  China | Prevention | Therapist | Provide health education to pregnant women on adverse emotions in late pregnancy and childbirth, and assist in coping with adverse conditions in late pregnancy and simulated childbirth training through different simulation scenarios. | Face-to-face, group-based | Health education: 3; Situation simulation visits: 4 | 60-90 mins | Health education: Once every four weeks;  Situation simulation visits: weekly | 8 weeks | T0: Baseline  T1: Post-delivery |
| Li, 2022  China | Prevention | Therapist | Damage and risk factors of adverse mental health (anxiety, depression and fear), identification of negative psychological issues, mood management, education for partner, childbirth, and peer support | Online | NR | 60 mins | NR | NR | T0: Baseline  T1: Post-intervention (2 weeks before childbirth)  T3: Follow up (1 week postpartum) |
| Li, 2023  China | NR | Therapist | Understanding negative emotion; cognition reconstruction: identifying wrong cognitions and changing wrong thinking inertia; cognitive reinforcement; reinforcing correct cognitions; behavioral modification: guiding new thinking patterns by learning correct cognition; behavioral reinforcement: reinforcing new behavioral patterns and introducing de-escalation methods. | Face-to-face, group-based | NR | NR | Weekly | NR | T0: Baseline  T1: Post-intervention |
| Liao, 2014  China | NR | Therapist | Controlling and avoiding maternal stressors; changing maternal cognitive appraisal; actively seeking family support; relaxation training. | Face-to-face, group-based | NR | NR | NR | NR | T0: Baseline  T1: Post-intervention |
| Lönnberg, 2021  Sweden | Prevention | Therapist | antenatal education was interwoven with mindfulness practices such as body scan, sitting and walking meditation, mindful movement, loving-kindness meditation, and informal meditation in daily life | Face-to-face, group-based | 8 | 135 mins | Weekly | 8 weeks | T0: Baseline  T1: Post-intervention  T2: 3-month postpartum  T3: 9-month postpartum  T4: 12-month postpartum |
| Loughnan, 2018  Australia | Treatment | Self-help | Psychoeducation about depression and anxiety, controlled breathing, progressive muscle relaxation; Psychoeducation about thoughts and unhelpful thoughts, through challenging, coping cards, structured problem-solving; Psychoeducation about unhelpful behaviors, activity planning, graded exposure, assertive communication, relapse prevention | Online | 3 | NR | Weekly | 4 weeks | T0: Baseline  T1: Post-intervention  T2: 4-week follow up |
| Lowndes, 2018  Australia | Prevention | Self-help | Psychoeducation about changing perfectionism and behavioral experiments to test perfectionism beliefs. Challenging unhelpful thinking styles, self-evaluation and relapse prevention | Not applicable | NR | NR | NR | 4 weeks | T0: Baseline  T1: Post-intervention  T2: 3-month follow-up(postpartum) |
| Lund, 2020  South Africa | Treatment | Therapist | Sessions included psychoeducation, problem-solving, behavioral activation, healthy thinking, relaxation training, and birth preparation. | Face-to-face | 6 | 45-60 mins | Weekly | 6 weeks | T0: Baseline  T1: Post-intervention (8-month gestation)  T2: 3-month postpartum  T3: 1-year postpartum |
| MacKinno, 2021  Canada | Prevention | Therapist | Participants were guided to recognize and disengage from unhelpful and ruminative thought patterns. They also received instruction in scheduling mastery and pleasant events and practice in mindful, assertive communication. | Face-to-face, group-based | 8 | 120 mins | Weekly | 8 weeks | T0: Baseline  T1: Post-intervention  T2: 3-month postpartum |
| Mahmoudi, 2023  Iran | Prevention | Therapist | (1) Anatomical and physiological changes in pregnancy and relaxation training; (2) stages of fetal development, performing attachment behaviors; (3) principles of nutrition and relaxation training through imagination about the fetus | Face-to-face, group-based | 3 | 90 mins | Every 3 days | NR | T0: Baseline  T1: Post-intervention |
| Mao, 2012  China | Prevention | Therapist | (1) understanding self-management and Chinese delivery culture; (2) effective problem solving and positive communication; (3) relaxation exercise and cognitive restructuring; (4) improving self-confidence | Face-to-face, group-based | 4 | 90 mins | Weekly | 4 weeks | T0: Baseline  T1: Post-intervention  T2: 6-week postpartum |
| Mao, 2021  China | Prevention | Therapist | Psychoeducation about antenatal care and childbirth, mental health issues, mood regulation and management; Peer support | Online | NR | NR | NR | NR | T0: Baseline  T1: Post-intervention |
| Mckee, 2006  China | Prevention | Therapist | (1) 8-session manualized CBT course covers topics such as cognitive distortions and reframing, relaxation techniques, increasing activities, social support and goal setting; (2) 4 child-development psychoeducational modules that focus on infant development and sensitive, responsive mothering | Face-to-face | 12 | NR | NR | NR | T0: Baseline  T1: Post-intervention |
| Mei, 2023  China | Treatment | Therapist | CBT: the therapy pays special attention to the specific sources of anxiety in pregnant women and uses a variety of techniques, such as cognitive restructuring, relaxation training, and coping strategy instruction, to help them manage and alleviate their anxiety;  Physical exercise tailored to women in the early to mid-stages of pregnancy, which includes low-intensity aerobic exercises (such as yoga and Tai Chi) and gentle resistance training | Face-to-face | 8 | NR | NR | NR | T0: Baseline  T1: Post-intervention  T2: 6-week postpartum |
| Missler, 2020  Netherlands | Prevention | Therapist | Sensitive responsiveness, adapting to the parental role, attending to own needs, crying patterns, feeding (arrangements), sleeping (arrangements) | Online | NR | 15-20 mins | NR | NR | T0: Baseline  T1: Post-intervention (34-36weeks’ gestation)  T2: 2weeks postpartum)  T3: 6 weeks postpartum  T4: 10 weeks postpartum |
| Mokaberian, 2021  Iran | Prevention | Therapist | In order to be physically relaxed, women were given a comfortable position and then were asked to start exercises, which included deep and slow abdominal breathing to bring about concentration. Then, relaxation was done by the use of mental imagery, which means that the mother was asked to imagine a scene in which she felt safe relieved of any stress and anxiety. | Face-to-face | 16 | 30 mins | Twice a week | 8 weeks | T0: Baseline  T1: Post-intervention |
| Montazeri, 2020  Iran | Prevention | Therapist | The concept of anxiety and factors affecting it, as well as disadvantages of anxiety and its impact on mothers; list anxiety-inducing factors and their worries on a piece of paper, and write their solutions in front of each factor | Face-to-face, group-based & telephone | 3 group writing therapy sessions and two telephone sessions | NR | writing therapy: biweekly | 6 weeks | T0: Baseline  T1: Post-intervention |
| Mortazavi, 2021  Iran | Prevention | Therapist | Help them formulate their goals based on the solution-focused approach; help identify the positive points of pregnancy and childbirth; help them identify appropriate ways of thinking, feeling and behavior. | Face-to-face | 5 | 60 mins | Weekly | 5 weeks | T0: Baseline  T1: Post-intervention |
| Mu, 2014  China | NR | Therapist | Psychological support; cognition intervention: explaining the etiology, pathology, physiology, development and regression of GDM and correcting misperceptions; behavioral intervention: explanation of the precautions for dietary treatment; follow-up management | Face-to-face, group-based | NR | NR | NR | NR | T0: Baseline  T1: Post-intervention |
| Muthukrishnan, 2016  India | Prevention | Therapist | The therapist explained the basics and techniques of mindfulness meditation, and women need to practice 30 minutes daily at home | Face-to-face, group-based | 10 | NR | Twice a week | 5 weeks | T0: Baseline  T1: Post-intervention |
| Nazari, 2018  Iran | Prevention | Therapist | (1) Definition of stress; (2) Optimism and its stages and benefits; (3) The techniques of reducing negative emotions and avoiding anxieties; (4) Social enhancement techniques; (5) living at present; (6) promoting creativity; (7) better planning, and their impact on happiness; (8) overview | Face-to-face, group-based | 8 | 90 mins | Twice a week | 4 weeks | T0: Baseline  T1: Post-intervention |
| Nejad, 2021  Iran | Prevention | Therapist | The main content of the MBSR program was standard yoga, sitting, walking, breathing, body scan and eating meditation. | Face-to-face, group-based | 8 | 120 mins | Weekly | 8 weeks | T0: Baseline  T1: Post-intervention |
| Ngai, 2022  Hong Kong | Prevention | Therapist | An overview of the changes associated with the role transition of parenthood; an introduction of the coping skills relating to role transition and parenting; a discussion of common postpartum and interpersonal difficulties. | Face-to-face, group-based | 3 | 120 mins | Weekly | 3 weeks | T0: Baseline  T1: 6-week postpartum;  T2: 6-month postpartum |
| Nishi, 2022  Japan | Prevention | Self-help | Psychoeducation, case formulation based on a cognitive-behavioral model, behavioral activation, self-compassion, mindfulness, problem-solving | Online | 6 | 5 mins | Weekly | 12-16 weeks | T0: Baseline  T1: Post-intervention (32 weeks’ gestation)  T2: 1 week postpartum  T3: 3-month postpartum |
| Pan, 2019  Taiwan^a, b^ | Prevention | Therapist | Teaching how to monitor self-sensory and emotional states and cognitive processes, deepen self-awareness, and become more mindful of the process of labor and parenting | Face-to-face | 8 classes and 1 day of 7 hours of silent meditation | 3 h | weekly | 8 weeks | T0: Baseline  T1: Post-intervention  T2: 36-week of gestation follow-up  T3: 3-months postpartum |
| Pan, 2023  Taiwan | Treatment | Therapist | Using mindfulness techniques such as meditation, body scan, breathing and so on to coping pregnancy and childbirth.  The specific contents of this course include awareness of fetal/newborn, mindfulness practices for improved interpersonal relationships, techniques for coping with labor pains, and women with PMAD how to adapt to perinatal life. | Face-to-face, group-based | 8 | 120 mins | Weekly | 8 weeks | T0: Baseline  T1: Post-intervention  T2: antenatal follow-up (36^th^ week)  T3: 2-month postpartum follow-up  T4: 4-month postpartum follow-up |
| Pinar, 2017  Turkey | Prevention | Therapist | It consists of information about stress, factors causing stress in pregnancy, coping with stress, breathing and muscle relaxation exercises. The stress management techniques and breathing and muscle relaxation exercises were taught using demonstration and role-play techniques | Face-to-face | 3 | 20-30 mins | Once every four weeks | 8 weeks | T0: Baseline  T1: Mid-study (4-week after baseline)  T2: Post-intervention |
| (1) Ponting, 2022  USA  (2) Urizar, 2019  USA | Prevention | Therapist | Teach women coping and relaxation skills for common prenatal stressors: stress awareness, thought monitoring and restructuring, coping skills, relaxation techniques, social support, and assertive communication | Face-to-face | 8 | 120 mins | weekly | 8 weeks | T0: Baseline  T1: Post-intervention  T2: 3^rd^ trimester follow-up (30-32 gestational period)  T3: 3-month postpartum |
| Puertas-Gonzalez, 2022  Spain | Prevention | Therapist | CBT: psychoeducation, relaxation, cognitive restructuring, alternative thought control, social skill training, emotional self-regulation, good mood and optimism;  psychological support: psychoeducation of stress in pregnancy, anxiety and depression | Online | 8 | 90-120 mins | weekly | 8 weeks | T0: Baseline  T1: Post-intervention |
| Ren, 2021  China | Treatment | Therapist | Therapeutic period: guiding the woman to visualize the event or situation that produced the bad mood and letting her notice and reflect on the irrational thoughts that arose. Correct irrational thoughts and reinforce positive ideas; relaxation training and learning correct problem-coping skills;  Consolidation period: online WeChat group discussion once a week | Online | 6 | 40-60 mins | Weekly | 6 weeks of treatment and 3 weeks of consolidation | T0: Baseline  T1: Post-intervention  T2: 3-day postpartum |
| Richter, 2012  Germany | Treatment | Therapist | The program consisted of psychoeducation, introduction to cognitive-behavioral strategies, performance of exercises/role-playing and progressive muscle relaxation. | Face-to-face, group-based | 8 | 90 mins | NR | NR | T0: Baseline  T1: Post-intervention  T2: 3-month postpartum |
| Romero-Gonzalez, 2020  Spain | Prevention | Therapist | (1) psychoeducation: what stress is, characteristics, identification of stressors, responses and consequences; (2) deactivation techniques (thematic imagination along with diaphragmatic breathing); (3) cognitive restructuring: cognitive distortions; (4) cognitive restructuring: irrational beliefs; (5) Alternative thought control strategies - self-instructional training and time organization; (6) training in social skills: assertiveness, basic assertive rights, saying no and asking for a change of behavior; (7) Relationship between anger and stress: emotional self-regulation; (8) optimism and good humor–recapitulation | Face-to-face, group-based | 8 | 90-120 mins | Weekly | 8 weeks | T0: Baseline  T1: Post-intervention |
| Rouhe, 2015  Finland | Treatment | Therapist | The focus of the intervention was to share the complex emotions and fears concerning the delivery and increase the feelings of safety and trust among the participants. Another aim was to strengthen the participants’ confidence regarding childbirth and motherhood and to increase their knowledge of the stages of delivery. | Face-to-face, group-based | 6 | 120 mins | NR | NR | T1: Baseline  T2: Post-intervention (1 month before delivery)  T3: 3-month postpartum |
| Sanaeinasab, 2020  Iran | Prevention | Therapist | Discussing the importance of childbearing and spiritual/religious rewards; Introduced the common psychological problems during pregnancy and presented the spiritual strategies for overcoming them; women were familiarized with material describing spiritual coping strategies related to using religious resources to deal with stress, anxiety and depression as recommended by the Holy Qur’an, Hadith, and other holy Islamic scriptures. | Face-to-face, group-based | 4 | 90 mins | Biweekly | 8 weeks | T0: Baseline  T1: 1-month after intervention  T2: 3-month after intervention |
| Scherer, 2016  Switzerland | Treatment | Therapist | The modules consisted of predetermined units (psychoeducational information, relaxation exercises) and interactive exercises (coping cards to reappraise stress-associated thoughts) as well as protocols (activity diary, stress and problem-solving protocol) | Online | 6 | NR | NR | NR | T0: Baseline  T1: Post-intervention |
| Shamabadi, 2023  Iran | Treatment | Therapist | (1) Auto-guidance; (2) focusing more on the body; (3) knowing how the mind can often be busy and distracted; (4) looking at events from a different angle in order to obtain a broad and different view of these events; (5) allowing oneself to be present, without judging it or trying to make a change;(6)instructed people about how negative moods and thoughts limit one’s connect with experience | Online | 6 | 90 mins | NR | 2 months | T0: Baseline  T1: Post-intervention  T2: 4-week follow-up |
| Smith, 2021  USA | Prevention | Self-help | Patients could use mobile apps (e.g., mindfulness meditation, sleep stories, nature sounds). Participants were encouraged to use the “daily Calm”, a 10-minute guided meditation unique to each day. | Online | NR | 10 mins | Daily | 30 days | T0: Baseline  T1: mid-study (14-day after baseline)  T2: Post-intervention (30-day after baseline) |
| Sun, 2021  China | Prevention | Self-help | Understand mindfulness; be in the present; be mindful of negative emotions; accept difficulties; thoughts are just thoughts; enjoy daily happiness; mindful pregnancy and childbirth; continue mindfulness practice (body scan, mindful breathing, mindful stretching, and mindful mediation) | Online | 8 | 25 mins | Weekly | 8 weeks | T0: Baseline  T1: Mid-study (4weeks after allocation)  T3: Post-intervention (8 weeks after allocation)  T4: Follow up (18weeks after allocation-before childbirth)  T5: 6 weeks postpartum Follow up |
| Surkan, 2023  Pakistan | Prevention | Self-help | It used the same core principles and strategies of the Thinking Healthy Program (THP), including developing empathetic relationships, thought challenging, behavior activation, problem management and enhancing family involvement and support | Face-to-face | 6 core sessions plus 6 supplementary booster sessions | NR | core sessions: weekly | NR | T0: Baseline  T1: 6-week postpartum |
| Tessema, 2024  Ethiopia | Prevention | Therapist | In the intervention, baby blues and postpartum depression, symptoms of PPD and when to recognize them, prevention methods, and the development of social support were included. | Face-to-face, group-based | 5 | 60-90 mins | Weekly | 5 weeks | T0: Baseline  T1: Post-intervention (6-week after delivery) |
| Toohill, 2014  Australia | Prevention | Therapist | The intervention aims to review women’s current expectations and feelings around fear of childbirth, support the expression of emotions, and provide a framework for women to identify and work through distressing elements of childbirth. | Telephone | 2 | NR | NR | 2 weeks | T0: Baseline  T1: Post-intervention (36 weeks’ gestation) |
| Vakilian, 2019  Iran | Prevention | Therapist | (1) Welcome and introduction; (2) living in the present and mindfulness; (3) experimental avoidance; (4) acceptance; (5) defusion; (6) self as context; (7) values; (8) committed action | Face-to-face | 8 | 90 mins | Twice a week | 4 weeks | T0: Baseline  T1: Post-intervention  T2: 4-week Follow up |
| Wu, 2023  China | Treatment | Therapist | Cognition intervention: constructing an online intervention platform, recording 6 courses related to perinatal mental health and childbirth knowledge, course training, communication and feedback; Behavior intervention: offline relaxation training and situational simulation health education exercise | Online | 6 cognition sessions and 3 behavior sessions | Cognition session: 40-60mins; Behavior session: 90mins | Weekly | 9 weeks | T0: Baseline  T1: Post-intervention |
| Yan, 2024  China | Prevention | Therapist | Provide health education to pregnant women on adverse emotions during pregnancy and the natural childbirth process, and improve the client’s ability to cope with various problems that may arise during childbirth through videos and situational simulations. | Face-to-face, group-based | 2-3 | 60 mins | NR | NR | T0: Baseline  T1: Before-delivery  T2: Post-delivery |
| Yang, 2019  China | Prevention | Therapist | Pay attention to the intended target and discrimination present experiences;  Adopting an accepting attitude toward physical and emotional experiences | Online | 4 | 40 mins | Biweekly | 8 weeks | T0: Baseline  T1: Post-intervention |
| Yazdanimehr, 2016  Iran | Treatment | Therapist | Overview of this program, basic principles of mindfulness and CBT, mindfulness breathing, behavior therapy, interpersonal skills, acceptance and management of suffering in daily life | Face-to-face, group-based | 8 | 90 mins | Weekly | 8 weeks | T0: Baseline  T1: Post-intervention  T2: 4-week follow-up |
| Zareneiad, 2020  Iran | Prevention | Therapist | Explain mindfulness and its mechanism; practicing awareness, breathing, relaxation and mediation techniques; explanation of fearfulness with pregnancy; talk about the needs of the neonate | Face-to-face, group-based | 6 group counseling session & weekly counselling | 60 mins | Twice a week | 3 weeks | T0: Baseline  T1: Post-intervention  T2:4-week follow-up |
| Zemestani, 2019  Iran | Treatment | Therapist | Included a stronger emphasis on brief formal and informal mindfulness and meditation practices customized for the perinatal period. Formal practices included the body scan, mindful yoga, and sitting meditation. Informal mindfulness practices included mindfulness of everyday activities such as mindful eating and walking. | Face-to-face, group-based | 8 | 120 mins | Weekly | 8 weeks | T0: Baseline  T1: Post-intervention  T2:4-week follow-up |
| Zhang, 2018  China | Prevention | Therapist | Approaching mindfulness; focus on your breath, open mindfulness; Move legs, keep mindfulness; Body scan; General yoga, nourish mindfulness; Sublimate mindfulness –Sitting Mouna; Incorporate mindfulness into life. | Face-to-face, group-based | 8 | 90 mins | Weekly | 8 weeks | T0: Baseline  T1: Post-intervention |
| Zhang, 2022  China | Treatment | Self-help | (1) introduction to mindfulness, (2) beyond automatic navigation, (3) becoming aware of the present experience, (4) identifying avoidance responses and accepting difficulties, (5) embracing happiness, and (6) mindfulness for childbirth. | Online | 6 | 10-20 mins | Weekly | 6 weeks | T0: Baseline  T1: Post-intervention |
| Zhang, 2023  China | Treatment | Self-help | Each module consisted of thematic lessons and homework. Each video was 10- to 20-minute long and included various cartoon images and mindfulness practice demonstrations. Homework, including formal and informal practices | Online | 6 | 10-20 mins | Weekly | 6 weeks | T0: Baseline (12-20weeks)  T1: Post-intervention (20-28weeks)  T2: Follow up (36-37weeks)  T3: 6-week postpartum Follow-up  T4: 3-month postpartum Follow up  T5: 6-month postpartum Follow up |
| Zhang, 2023  China | Prevention | Self-help | The mindfulness courses included mindfulness-based stress reduction, some components of mindfulness-based cognitive therapy, and acceptance and commitment therapy. Further, the courses incorporated the experience of pregnancy and childbirth. Mindfulness practices refer to classic formal and informal meditation, such as a body scan and mindful breathing. | Online | 4 | 30 mins sessions and 30-45 mins per day of mindfulness practice | Weekly | 4 weeks | T0: Baseline  T1: Post-intervention  T2: 15-week follow-up |

Note: NR=Not reported; Mins=Minutes; APP=Application
