# Supplementary material for: Effects of prenatal psychotherapies and psychosocial interventions on depressive symptoms, anxious symptoms and stress: a systematic review and network meta-analysis
Source: Front Psychiatry. 2026 Jan 28;16:1624924. doi: 10.3389/fpsyt.2025.1624924 (PMC12890675; doi:10.3389/fpsyt.2025.1624924)
Supplement: Supplementary file 1 [file DataSheet1.zip › 新建文件夹/Supplementary Table 3. The P-score derived from the SUCRA for each psychological symptom.docx]

Supplementary Table 3. The P-score derived from the SUCRA for each psychological symptom

| Treatments | Depression | Anxiety | Stress |
| --- | --- | --- | --- |
| Multicomponent interventions | **0.934** | 0.310 | **1.000** |
| IPT | 0.639 | 0.731 | 0.239 |
| MBI | 0.621 | 0.590 | 0.754 |
| CBT | 0.571 | 0.549 | 0.622 |
| Psychoeducation | 0.191 | 0.172( | 0.261 |
| Counseling | - | 0.739 | 0.520 |
| ACT | - | **0.875** | - |
| Control | 0.043 | 0.036 | 0.113 |

Note: IPT= Interpersonal treatment; CBT=Cognitive behavioral therapy; MBI=Mindfulness-based intervention; ACT=Acceptance and commitment therapy; the bold figures indicate the highest values.
