# Supplementary material for: Effects of prenatal psychotherapies and psychosocial interventions on depressive symptoms, anxious symptoms and stress: a systematic review and network meta-analysis
Source: Front Psychiatry. 2026 Jan 28;16:1624924. doi: 10.3389/fpsyt.2025.1624924 (PMC12890675; doi:10.3389/fpsyt.2025.1624924)
Supplement: Supplementary file 1 [file DataSheet1.zip › 新建文件夹/Supplementary Table 4. The result of meta-regression analyses.docx]

Supplementary Table 4. The result of meta-regression analyses

(a) The results of meta-regression analyses for depression scores

| Predictor | CBT vs control | | MBI vs control | | | Multicomponent vs control | | Psychoeducation vs control | |
| --- | --- | --- | --- | --- | --- | --- | --- | --- | --- |
|  | Coef(SE) | p | Coef(SE) | | p | Coef(SE) | p | Coef(SE) | p |
| Publication year |  |  |  | |  |  |  |  |  |
| ≥2020 | 0.25(0.38) | 0.53 | 0.73(0.72) | | 0.33 | 0.35(1.07) | 0.75 | 0.02(0.12) | 0.82 |
| <2020 | ref | | ref | | | ref | | ref | |
| Geographic region |  | |  | | |  | |  | |
| Asia | **-0.75(0.33)** | **0.04** | -0.81(0.68) | 0.26 | | -1.51(1.14) | 0.21 | -0.06(0.10) | 0.56 |
| Non-Asia | ref | | ref | | | ref | | ref | |
| Intervention platform |  | | | | | | | | |
| Online | -0.90(0.48) | 0.12 | 1.42(1.24) | | 0.29 | 1.61(1.65) | 0.38 | 0.10(0.14) | 0.54 |
| Face-to-face | ref | | ref | | | ref | | ref | |
| Purpose |  | | | | | | | | |
| Treatment | -0.82(0.41) | 0.10 | -0.96(0.83) | | 0.29 | 2.21(1.81) | 0.28 | 0.26(0.40) | 0.58 |
| Prevention | ref | | ref | | | ref | | ref | |
| Guidance status |  | | | | | | | | |
| Therapist-assisted | 0.16(0.53) | 0.77 | -0.36(1.13) | | 0.76 | - | | - | |
| Self-help | ref | | ref | | | - | | - | |

Note: CBT= Cognitive behavioral therapy; MBI= Mindfulness-based intervention; ref=reference; Coef=Coefficient; SE=Standard error.“-”: insufficient observations

(b) The results of meta-regression analyses for anxiety scores

| Predictor | CBT vs control | | MBI vs control | | Multicomponent vs control | | Psychoeducation vs control | |
| --- | --- | --- | --- | --- | --- | --- | --- | --- |
|  | Coef(SE) | p | Coef(SE) | p | Coef(SE) | p | Coef(SE) | p |
| Publication year |  |  |  |  |  |  |  |  |
| ≥2020 | -0.27(0.33) | 0.42 | 0.86(0.53) | 0.14 | **1.14(0.30)** | **<0.01** | -0.22(0.25) | 0.45 |
| <2020 | ref | | ref | | ref | | ref | |
| Geographic region |  | |  | |  | |  | |
| Asia | **-0.85(0.24)** | **0.004** | -0.82(0.60) | 0.20 | -0.32(2.03) | 0.88 | -0.08(0.29) | 0.80 |
| Non-Asia | ref | | ref | | ref | | ref | |
| Intervention platform |  | | | | | | | |
| Online | 1.46(8.17) | 0.87 | 8.71(3.20) | 0.04 | -1.68(3.83) | 0.68 | 3.57(1.82) | 0.30 |
| Face-to-face | ref | | ref | | ref | | ref | |
| Purpose |  | | | | | | | |
| Treatment | 8.34(7.27) | 0.30 | -2.54(2.35) | 0.33 | -5.06(3.91) | 0.27 | - | |
| Prevention | ref | | ref | | ref | | - | |
| Guidance status |  | | | | | | | |
| Therapist-assisted | 6.36(10.25) | 0.56 | -0.53(2.88) | 0.86 | - | | - | |
| Self-help | ref | | ref | | - | | - | |

Note: CBT=Cognitive behavioral therapy; MBI=Mindfulness-based intervention; ref=reference; Coef=Coefficient; SE=Standard error;“-”: insufficient observations

(c) The results of meta-regression analyses for stress scores

| Predictor | CBT vs control | | | MBI vs control | |
| --- | --- | --- | --- | --- | --- |
|  | Coef(SE) | p | | Coef(SE) | p |
| Publication year |  |  | |  |  |
| ≥2020 | -0.15(0.46) | 0.76 | | **2.96(1.20)** | **0.04** |
| <2020 | ref | | | ref | |
| Geographic region |  | | | | |
| Asia | **-1.05(0.30)** | **<0.01** | -1.19(1.57) | | 0.47 |
| Non-Asia | ref | | ref | | |
| Intervention platform |  | | | | |
| Online | 1.01(0.46) | 0.05 | | 10.07(4.22) | 0.09 |
| Face-to-face | ref | | | ref | |
| Purpose |  | | | | |
| Treatment | 0.55(0.47) | 0.27 | | - | |
| Prevention | ref | | | - | |
| Guidance status |  | | | | |
| Therapist-assisted | - | | | -1.24(3.55) | 0.75 |
| Self-help | - | | | ref | |

Note: CBT=Cognitive behavioral therapy; MBI= Mindfulness-based intervention; ref=reference; Coef=Coefficient; SE=Standard error;“-”: insufficient observations
