# Supplementary material for: Effects of prenatal psychotherapies and psychosocial interventions on depressive symptoms, anxious symptoms and stress: a systematic review and network meta-analysis
Source: Front Psychiatry. 2026 Jan 28;16:1624924. doi: 10.3389/fpsyt.2025.1624924 (PMC12890675; doi:10.3389/fpsyt.2025.1624924)
Supplement: Supplementary file 1 [file DataSheet1.zip › 新建文件夹/Supplementary Table 5. The results of certainty of evidence assessment.docx]

Supplementary Table 5. The results of certainty of evidence assessment

(1) The results of certainty assessment for depressive symptoms

| Comparison | Number of studies | Within-study bias | Reporting bias | Indirectness | Imprecision | Heterogeneity | Incoherence | Confidence rating |
| --- | --- | --- | --- | --- | --- | --- | --- | --- |
| CBT vs Control | 15 | Some concerns | No concerns | No concerns | No concerns | Major concerns | No concerns | Low |
| MBI vs Control | 13 | Some concerns | No concerns | No concerns | No concerns | Major concerns | No concerns | Low |
| Multicomponent interventions vs Control | 12 | Some concerns | No concerns | No concerns | No concerns | Major concerns | No concerns | Low |
| Psychoeducation vs control | 7 | Some concerns | No concerns | No concerns | No concerns | No concerns | No concerns | Moderate |
| IPT vs control | 4 | Some concerns | No concerns | No concerns | No concerns | Major concerns | No concerns | Low |
| CBT vs Psychoeducation | 1 | Some concerns | No concerns | Some concerns | Major concerns | No concerns | No concerns | Low |

Note: CBT=Cognitive behavioral therapy; MBI=Mindfulness-based intervention; IPT= Interpersonal treatment.

(2) The results of certainty assessment for anxious symptoms

| Comparison | Number of studies | Within-study bias | Reporting bias | Indirectness | Imprecision | Heterogeneity | Incoherence | Confidence rating |
| --- | --- | --- | --- | --- | --- | --- | --- | --- |
| CBT vs Control | 15 | Some concerns | No concerns | No concerns | No concerns | Major concerns | No concerns | Low |
| MBI vs Control | 12 | Some concerns | No concerns | No concerns | No concerns | Major concerns | No concerns | Low |
| Multicomponent interventions vs Control | 9 | Some concerns | No concerns | No concerns | Some concerns | Major concerns | No concerns | Low |
| Psychoeducation vs control | 5 | Some concerns | No concerns | No concerns | Some concerns | Some concerns | No concerns | Low |
| Counseling vs control | 4 | Some concerns | No concerns | No concerns | No concerns | Some concerns | No concerns | Low |
| IPT vs control | 1 | Some concerns | No concerns | No concerns | Major concerns | No concerns | No concerns | Moderate |
| ACT vs control | 1 | Some concerns | No concerns | No concerns | Some concerns | No concerns | No concerns | Low |

Note: CBT=Cognitive behavioral therapy; MBI=Mindfulness-based intervention; IPT=Interpersonal treatment; ACT=Acceptance and commitment therapy.

(3) The results of certainty assessment for stress

| Comparison | Number of studies | Within-study bias | Reporting bias | Indirectness | Imprecision | Heterogeneity | Incoherence | Confidence rating |
| --- | --- | --- | --- | --- | --- | --- | --- | --- |
| CBT vs Control | 11 | Some concerns | No concerns | No concerns | No concerns | Major concerns | No concerns | Low |
| MBI vs Control | 9 | Some concerns | No concerns | No concerns | No concerns | Major concerns | No concerns | Moderate |
| Multicomponent interventions vs Control | 1 | Some concerns | No concerns | No concerns | No concerns | No concerns | No concerns | Moderate |
| Psychoeducation vs control | 4 | Some concerns | No concerns | No concerns | Major concerns | Some concerns | No concerns | Low |
| Counseling vs control | 2 | Some concerns | No concerns | No concerns | No concerns | Some concerns | No concerns | Low |
| IPT vs control | 1 | Some concerns | No concerns | No concerns | Major concerns | No concerns | No concerns | Low |
| CBT vs Psychoeducation | 1 | Some concerns | No concerns | No concerns | Major concerns | No concerns | No concerns | Low |

Note: CBT=Cognitive behavioral therapy; MBI=Mindfulness-based intervention; IPT=Interpersonal treatment.
