# Supplementary figures and images for: Effects of prenatal psychotherapies and psychosocial interventions on depressive symptoms, anxious symptoms and stress: a systematic review and network meta-analysis
Source: Front Psychiatry. 2026 Jan 28;16:1624924. doi: 10.3389/fpsyt.2025.1624924 (PMC12890675; doi:10.3389/fpsyt.2025.1624924)

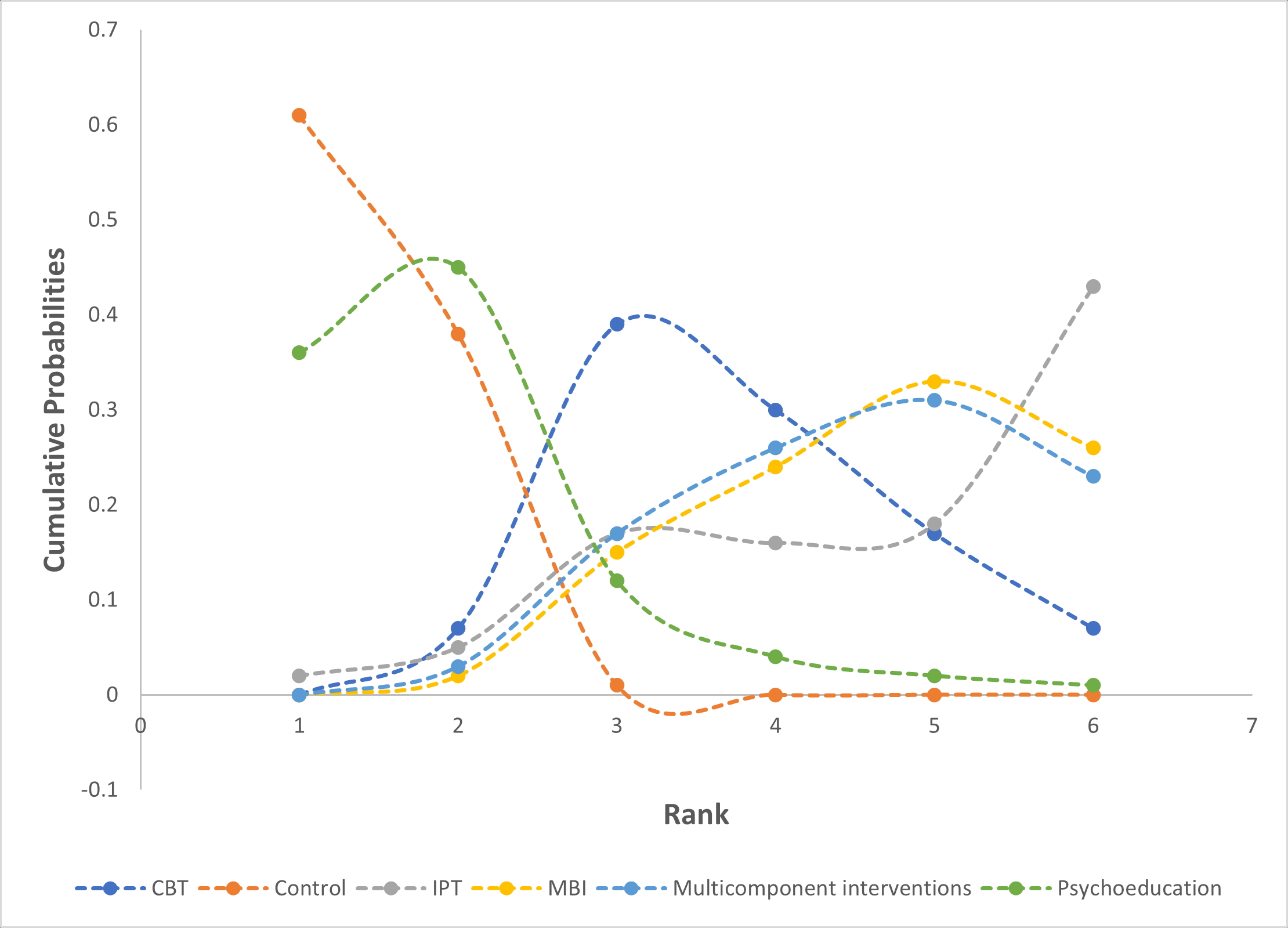

Supplement: Supplementary file 1 [file DataSheet1.zip › 新建文件夹/Supplementary Figure 1. The SUCRA curve of different interventions for depressive symptoms.jpg]

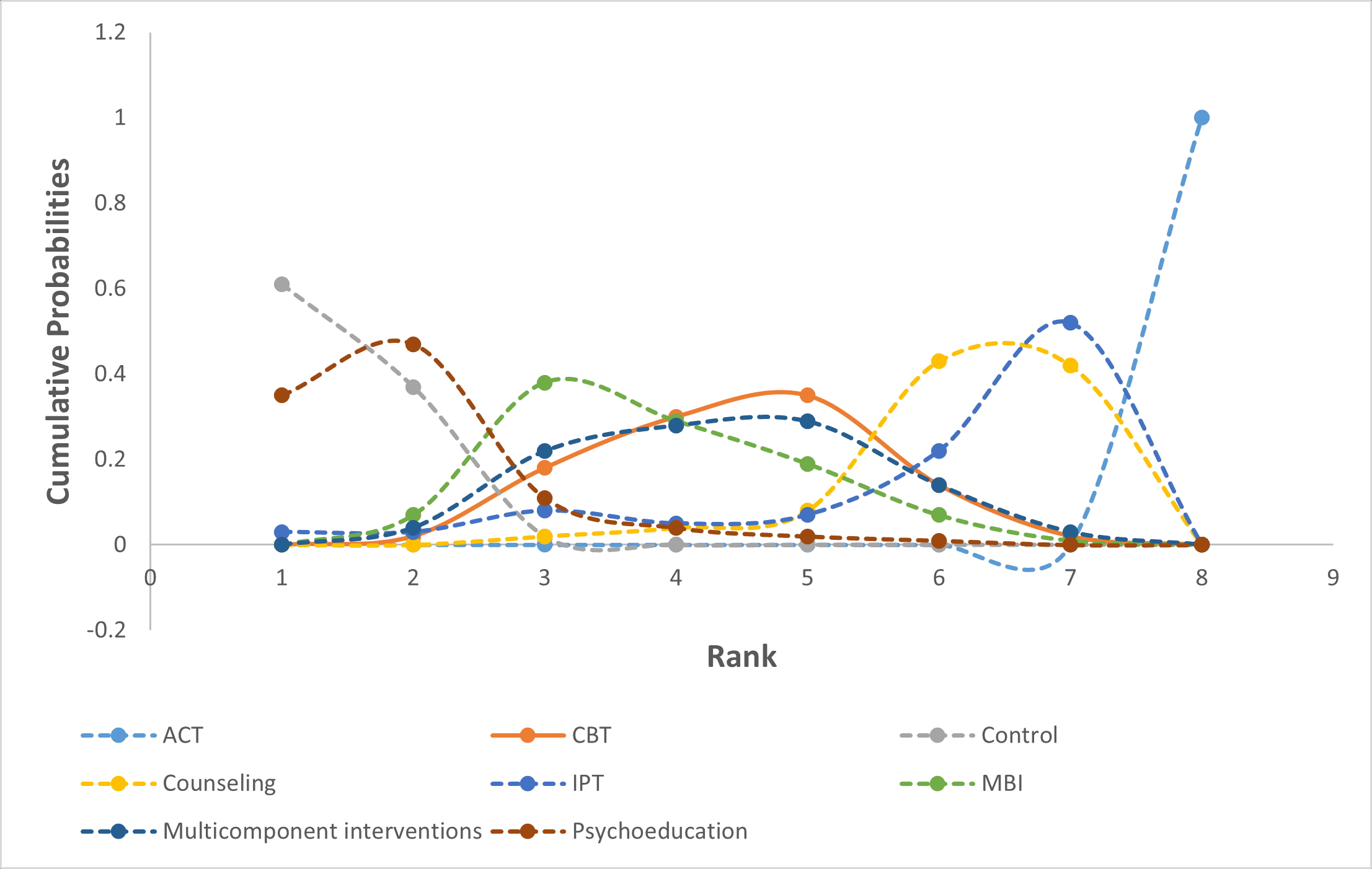

Supplement: Supplementary file 1 [file DataSheet1.zip › 新建文件夹/Supplementary Figure 2. The SUCRA curve of different interventions for anxious symptoms.jpg]

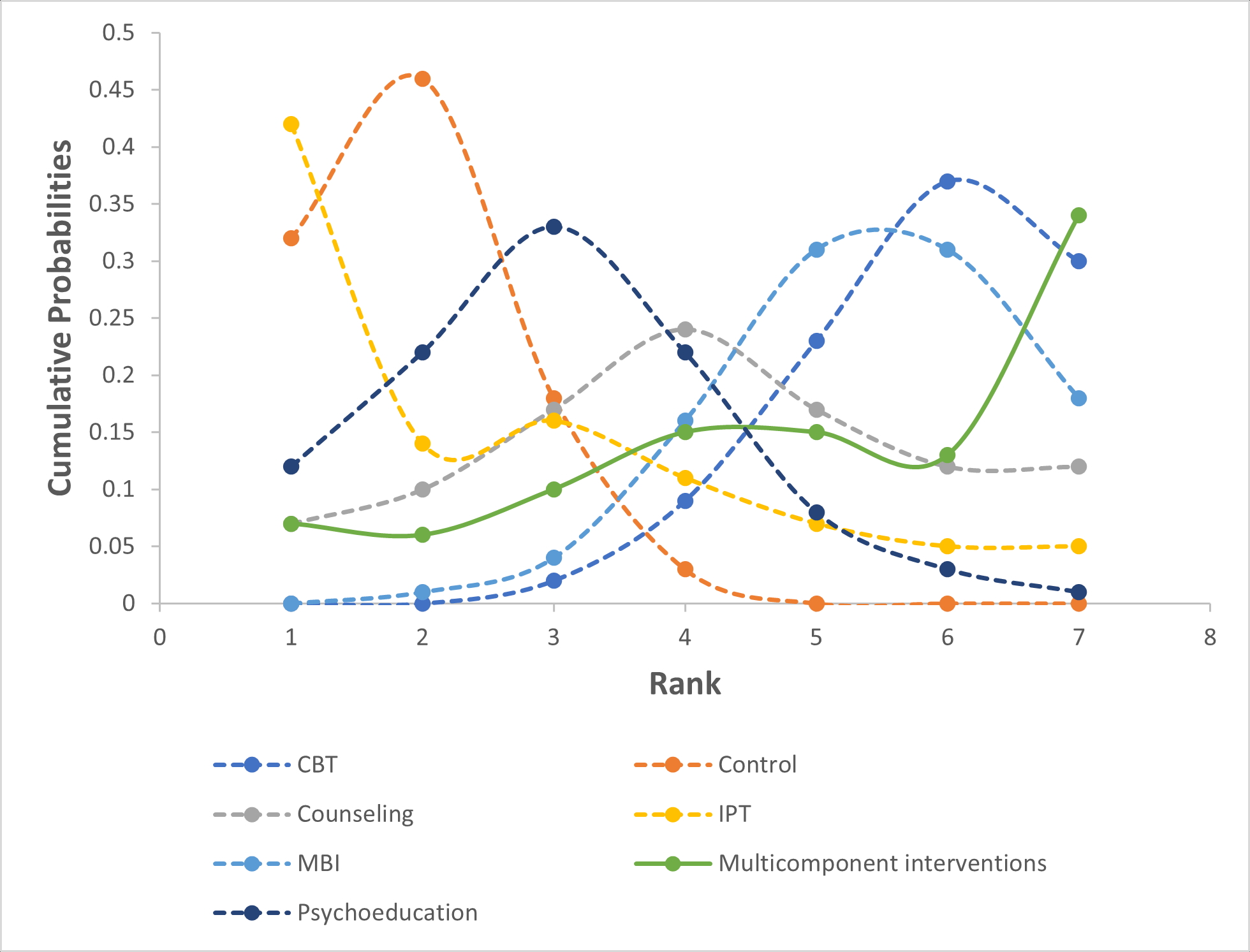

Supplement: Supplementary file 1 [file DataSheet1.zip › 新建文件夹/Supplementary Figure 3. The SUCRA curve of different interventions for stress.jpg]
